# A qualitative study on aviophobia and coping strategies

**DOI:** 10.1186/s40359-026-05050-y

**Published:** 2026-06-24

**Authors:** Eylül Eyiz, Kübra Nur Kırhan, Sevdiye Kemik Polat, Mustafa Aslan

**Affiliations:** 1 Faculty of Applied Sciences, Department of Aviation Management, Istanbul Bilgi University, Beyoglu, Istanbul, 34440 Türkiye; 2 Faculty of Economics, Administrative and Social Sciences, Department of Aviation Management, Istanbul Topkapi University, Istanbul, Türkiye

**Keywords:** Aviophobia, Fear of flying, Factors affecting aviophobia, Coping strategies

## Abstract

**Background:**

Fear of flying, or aviophobia, is one of the most prevalent phobias, affecting approximately 3 to 5% of the population. Although air travel has become significantly safer, fear of flying continues to impact individuals’ social, professional, and personal lives, often leading to avoidance behaviors and significant distress. This qualitative study explores the causes, triggers, and coping strategies associated with fear of flying, focusing on participants from Türkiye.

**Methods:**

Through semi-structured interviews with 20 individuals with self-reported fear of flying, conducted between June 2024 and February 2025, the study identifies key factors associated with the fear of flying, including psychological factors (e.g., anxiety disorders, claustrophobia), negative flight-related experiences (e.g., turbulence, media coverage of crashes), cognitive factors (e.g., catastrophic thinking), environmental and social influences (e.g., learned behaviors, media exposure), physiological responses (e.g., sweating, palpitations), and cultural contexts. Data were analyzed using thematic analysis, and data collection continued until thematic saturation was achieved.

**Results:**

The findings reveal that turbulence, fear of enclosed spaces, and media portrayals of aviation accidents are significant triggers. Coping strategies such as Cognitive-Behavioral Therapy (CBT), relaxation techniques, and spiritual methods (e.g., praying) are commonly employed to manage flight anxiety. The study also highlights the role of social media in relation to fear of flying, as exposure to negative aviation content is associated with increased anxiety and avoidance behaviors. Despite the availability of professional support and airline-sponsored programs, many participants reported a notable underutilization of these resources.

**Conclusions:**

The study concludes that a multifaceted approach, combining psychological interventions, education, and culturally sensitive treatments, may be important for addressing fear of flying. These findings contribute to the growing body of literature on fear of flying and provide insights for developing targeted interventions to help individuals overcome their fear of flying.

## Introduction

Fear of flying, also known as aerophobia or aviophobia, is among the top ten most prevalent phobias in society. It is defined in the Diagnostic and Statistical Manual of Mental Disorders, Fifth Edition (DSM-5-TR) as “*a marked and persistent fear or anxiety that is excessive or unreasonable*,* cued by the presence or anticipation of a specific object or situation*” [1]. Although aviation has advanced significantly and is considered the safest mode of transportation [2], with less than 2.5% accident rates per flight hour and fewer than 8% passenger fatalities [3], fear of flying persists as a psychological reaction to perceived risks, dating back to the early history of aviation [4].

While fear of flying is more commonly reported by females than males [5], it spans all socio-demographic groups. Approximately 3 to 5% of the global population avoids air travel due to fear [6]. The severity of fear of flying can vary—ranging from mild unease to severe panic—and it may adversely affect individuals’ professional functioning, social relationships [7], and physical well-being through symptoms such as increased heart rate, shortness of breath, or gastrointestinal distress [8].

Although fear of flying has been extensively studied in the past [8], the majority of research has focused on populations in Western Europe and the United States. Few studies explore the cultural, psychological, and behavioral dimensions of flight phobia within non-Western contexts. This study aims to address this gap by examining the causes, triggers, and coping strategies related to fear of flying among individuals in Türkiye. In doing so, it also seeks to compare these findings with existing literature and contribute a culturally nuanced understanding to the field. Accordingly, the focus of this study is not on evaluating therapeutic effectiveness or providing in-depth clinical explanations, but on qualitatively describing how individuals experience, interpret, and cope with their fear of flying.

This study is grounded in a biopsychosocial framework, which conceptualizes fear of flying as a multidimensional phenomenon shaped by the interaction of cognitive, emotional, environmental, and sociocultural factors. From this perspective, fear of flying is not reduced to a single cause but is understood as emerging from the dynamic interplay between individual vulnerabilities (e.g., anxiety sensitivity, cognitive distortions), situational triggers (e.g., turbulence, media exposure), and broader contextual influences (e.g., cultural beliefs and social learning processes). This approach is consistent with contemporary models of anxiety and specific phobia, which emphasize the role of cognitive appraisal, perceived lack of control, and learned associations in the development and maintenance of fear responses. Accordingly, the present study adopts an interpretive stance, focusing on how individuals construct and experience fear of flying within their lived contexts, rather than attempting to establish causal or diagnostic explanations.

### Causes of fear of flying

A phobia is an intense and disproportionate fear or anxiety when confronted with a particular situation, activity, organism, location, or object. It can lead individuals to change their lifestyles to avoid potential encounters with the triggering object or scenario, which can negatively impact daily functioning [8, 9]. Fear of flying can be seen as an effect of phobias such as claustrophobia, acrophobia [10], posttraumatic stress disorder (PTSD), or agoraphobia.

Although genetics is one of the factors associated with fear of flying [11], the present study focuses on experiential, cognitive, and contextual factors. Fear of flying is a multifaceted phenomenon, and although cognitive-behavioral perspectives dominate the literature, alternative theoretical frameworks offer complementary explanations.

From a psychodynamic perspective, specific phobias may represent symbolic manifestations of deeper unconscious conflicts, where the feared object (e.g., flying) serves as a displacement of broader anxieties such as loss of control or mortality concerns. In this sense, symptom-focused interventions may reduce flight-related fear, while underlying anxiety may shift to other domains.

From a relational and developmental perspective, early social environments and learned experiences play a critical role. Individuals raised in contexts where flying is absent or framed as dangerous may internalize these perceptions. Additionally, dependency patterns and fear of autonomy may intensify anxiety in situations involving a lack of control, such as air travel.

Therefore, fear of flying should be understood within a broader biopsychosocial framework that integrates cognitive, emotional, relational, and cultural dimensions rather than being reduced to a single explanatory model. Accordingly, the most common and key contributing factors, based on prior research and the data obtained in this study, are outlined below:

#### Psychological factors

These include underlying anxiety disorders [8, 9], fear of losing control [12], fear of heights [2], and claustrophobia [13]. In individuals with Generalized Anxiety Disorder (GAD), panic disorder, or other anxiety-related conditions, flying often triggers a loss of perceived safety and autonomy [14]. Emotional factors, such as powerlessness and lack of control, can exacerbate fear of flying in enclosed environments such as airplane cabins [15].

#### Negative flight-related experiences

Direct or secondhand exposure to negative flying experiences—such as turbulence, emergency landings, or witnessing accidents via media— may heighten fear [16, 17]. Individuals tend to recall negative flight experiences more vividly than positive ones, creating anticipatory anxiety and avoidant behavior. Media coverage may amplify the emotional impact of rare aviation disasters, making them appear more common than they are [18]. Notably, studies after major aviation incidents show long-lasting psychological consequences, including PTSD symptoms among indirectly exposed individuals [16].

#### Cognitive factors

Catastrophic thinking, intolerance of uncertainty, and the tendency to overestimate risk are common cognitive distortions associated with fear of flying [14, 19]. Individuals with low aviation literacy may interpret normal events—like turbulence—as signs of danger, reinforcing fear [2]. Additionally, misinterpretation of physiological symptoms (e.g., heart palpitations) can further escalate anxiety during flight [13].

#### Environmental and social factors

According to Social Learning Theory [19], individuals may develop phobias through observational learning—particularly from caregivers or peers [20]. Fear of flying can arise when flying is framed as dangerous in childhood or through repeated exposure to fear-inducing narratives [21]. The media—especially social media—may play a critical role in shaping and reinforcing fear, particularly when accident footage or panic episodes are widely circulated [22, 23].

#### Physiological factors

Bodily sensations experienced during flight—such as changes in cabin pressure, engine noise, or turbulence—may induce hyperarousal and panic in susceptible individuals [13]. These symptoms (e.g., rapid heartbeat, sweating, dizziness) often trigger the “fight or flight” response and are misinterpreted as signals of impending disaster. There is also evidence suggesting that neurochemical imbalances, particularly in serotonin and dopamine systems, may predispose individuals to phobic reactions, though this falls outside the scope of this study [24].

#### Cultural and contextual factors

Cultural values, religious beliefs, and local perceptions of fate, risk, or divine protection may influence how individuals interpret the threat of flying [2]. Life stressors such as bereavement, health crises, or job loss can also heighten emotional vulnerability and lower resilience, increasing the likelihood of phobic response [25]. In the Turkish context, individuals often turn to religious coping mechanisms in situations involving stress and uncertainty [26].

### Strategies for coping with fear of flying

Cognitive Behavioral Therapy (CBT) is widely used to cope with fear of flying. CBT aims to uncover and modify maladaptive cognitive processes related to flying, with the aim of reducing anxiety levels. Methods like systematic desensitization and cognitive restructuring are used to expose patients to the stimulus that causes anxiety in a controlled way, aiding them in developing coping strategies [14]. While CBT is one of the most widely utilized approaches in addressing fear of flying, it should not be considered the sole or definitive treatment, as a range of alternative therapeutic perspectives and coping strategies may also contribute to managing fear of flying, depending on individual differences and contextual factors.

Disseminating knowledge regarding the principles of aviation, safety data, and typical factors contributing to turbulence may help reduce anxiety. Acquiring knowledge decreases uncertainty and clarifies the flying experience, which may help alleviate unreasonable anxieties [8].

Various relaxation techniques, such as deep breathing exercises, progressive muscle relaxation, and mindfulness meditation, have been proven to be effective in alleviating symptoms of anxiety. These strategies assist individuals in regulating their physiological reactions to worry, facilitating a state of tranquility while traveling by air [27]. People frequently use relaxation techniques and breathing exercises to reduce anxiety during flights, which helps them manage stress levels and mitigate symptoms [28]. Medication can offer transient alleviation, particularly for severe anxiety, but it should be utilized alongside other therapeutic modalities for sustained relief [15].

Exposure treatment entails systematically and repeatedly exposing individuals to the feared situation, such as flying, in a controlled setting. This process helps reduce the individual’s sensitivity to anxiety-inducing stimuli. Virtual reality exposure treatment (VRET) is a highly effective method for treating fear of flying, as it recreates flight experiences in a secure and regulated environment [29].

Pharmacotherapy may be employed as a supplementary treatment to psychological therapies in certain instances. Temporary utilization of anxiolytics or beta-blockers can effectively control immediate anxiety symptoms seen during flights. Nevertheless, this method is generally seen as a final option because of the possibility of reliance and adverse consequences [7]. Individuals suffering from phobias may find books and websites that include cognitive-behavioral strategies, relaxation exercises, and educational information to be helpful, even without professional help [30].

Furthermore, some airlines offer support groups and fear-of-flying courses that give a communal setting for individuals to discuss their experiences and coping strategies. These programs typically consist of a blend of instructional sessions, therapeutic interventions, and supervised flights to enhance confidence and diminish fear [31]. Turkish Airlines’ “Overcoming Fear of Flying Program” [32] and Lufthansa Airlines’ seminars that involve psychologists and psychotherapists to assist passengers in overcoming their fear of flying [33] may be considered as examples of such efforts to overcome fear of flying.

## Methods

The qualitative study investigates the experiences of individuals who have fear of flying. The interviews were carried out face-to-face between June 2024 and February 2025, and open-ended questions about the causes and symptoms of fear of flying and coping strategies were asked. The responses were thematically analyzed and further subjected to analysis using MAXQDA 2024 software to reveal interconnections between factors and triggers of flight phobia, coping techniques, and psychological and physical symptoms elicited at times of flight.

The study followed the principles of qualitative rigor as proposed by Lincoln & Guba [34], ensuring credibility through triangulation, dependability through an audit trail, confirmability through independent coding, and transferability through thick description of participant experiences. This study was also reported in accordance with the Consolidated Criteria for Reporting Qualitative Research (COREQ) checklist. Reflexivity was maintained throughout via analytic memoing and team debriefing.

Furthermore, the use of frequency counts in this study is intended solely to provide descriptive support for the prominence of themes across participants, rather than to imply statistical generalizability. In line with qualitative research principles, thematic interpretation remains the primary focus of analysis, and numerical expressions are used only to enhance transparency and reader comprehension [35, 36].

Snowball sampling is a non-probability, network-based recruitment strategy particularly suitable for accessing hard-to-reach or otherwise difficult-to-identify populations where conventional sampling frames are not readily available [37]. In such designs, participants are recruited through existing social connections, facilitating access and increasing participation willingness due to interpersonal trust and social proximity [38, 39]. This approach is widely recognized in qualitative research as a pragmatic and theoretically justified strategy for accessing experiential knowledge in populations that are not easily identifiable through formal sampling frames, particularly when the research focus is on subjective experiences rather than population-level generalization [38, 39].

### Participants

Inclusion criteria were defined a priori as follows: (1) age 18 years or older; (2) self-reported fear of flying; and (3) prior flight experience within the last 12 months. Individuals who had never flown, or whose most recent flight was more than one year before the interview, were excluded, as the study aimed to capture recent, first-hand accounts of in-flight emotional, cognitive, and physiological responses. No formal diagnostic assessment was applied; fear of flying is used here in a spectrum-based, non-clinical sense consistent with prior qualitative literature [2].

A total of 20 adults (Table 1) aged 22–63 (M = 38.5) participated in the study. To access this relatively difficult-to-reach population, a controlled snowball sampling strategy was employed. Multiple independent referral chains were initiated, each capped at three links, to limit social homogeneity. Data collection continued until thematic saturation was reached, which was observed after approximately the 16th interview, with subsequent interviews confirming the stability of the coding structure [36, 40].

**Table 1:** Professions of the participants

Participant	Gender	Age	Occupation
P1	Male	22	Student
P2	Female	59	Housewife
P3	Female	38	Cleaning worker
P4	Male	60	Civil engineer
P5	Male	22	Student
P6	Male	62	Watch designer
P7	Male	55	Jeweler
P8	Female	58	Housewife
P9	Female	25	Sports Instructor
P10	Female	30	Secretary
P11	Female	55	Beautician
P12	Female	25	Student
P13	Male	30	Crane Operator
P14	Male	23	Student
P15	Male	32	Hairdresser
P16	Female	26	Fashion consultant
P17	Female	29	Nurse
P18	Female	23	Intern Lawyer
P19	Male	33	Academic
P20	Male	63	Retired

Although the sample size of 20 participants may appear limited from a quantitative perspective, it is consistent with qualitative research standards that prioritize depth over breadth. Data collection was guided by the principle of thematic saturation, defined as the point at which no new themes or meaningful insights emerge from additional data [36, 40]. Recurring patterns began to stabilize after approximately the 16th interview, with subsequent interviews confirming the stability of the coding structure rather than generating new themes. This is consistent with prior qualitative research suggesting that saturation in relatively homogeneous samples is often achieved within the first 12–20 interviews [40], particularly when the research focus is narrow and the participant group shares a common experiential domain. Furthermore, qualitative research does not aim for statistical representativeness but rather for analytical depth and conceptual richness; the sample of 20 was therefore considered sufficient to capture diverse experiential patterns while allowing for in-depth exploration of participants’ cognitive, emotional, and behavioral processes [41].

### Questions

The semi-structured interview protocol was revised to include open-ended, exploratory questions designed to elicit participants’ subjective experiences, emotional narratives, and meaning-making processes related to their fear of flying. Questions encouraged participants to reflect on the origins of their fear, bodily and cognitive responses, coping strategies, and the broader impact of fear of flying on their lives.

In this regard, 13 questions that are categorized under three main headings: Causes of Fear of Flying, Symptoms of Fear of Flying, and Ways to Cope with the Fear of Flying, were formulated and posed to participants to explore the fear of flying.

Causes of Fear of Flying

- Can you tell me about the first time you remember feeling fear related to flying? What do you think triggered it?
- How has your fear of flying changed over time? What keeps it going, in your opinion?
- Can you describe situations—before or during a flight—that make your fear worse?
- How do stories or news about aviation incidents affect your emotions or decisions about flying?
- Where do you usually hear about aviation-related accidents? How do these sources influence your thoughts about flying?

Symptoms of Fear of Flying

- Can you walk me through your emotional state during the time leading up to a flight? What thoughts or feelings do you experience?
- During the flight, what kinds of thoughts or emotions come up for you? Can you describe a recent experience?
- What kinds of physical sensations do you notice when you’re afraid during a flight? What do you think they mean to you?
- Are there particular parts of the flight that are more difficult than others (e.g., takeoff, turbulence)? What makes them harder for you?

Ways to Cope with the Fear of Flying

- What do you do to help yourself feel calmer or safer during a flight? How effective are those methods for you?
- What are the most distressing thoughts or images that enter your mind when you’re on a plane?
- How has your fear of flying influenced your life—your travel, your work, or your relationships?
- Have you ever considered or tried any support (therapy, medication, airline programs)? What influenced that decision?

### Findings and discussion

During the research, participants’ responses were analyzed and categorized by theme. Each theme was further divided into subcategories, enabling a more nuanced understanding of participants’ experiences with fear of flying.

Numerical expressions (e.g., percentages) are used in a limited and descriptive manner to illustrate the relative prominence of themes, rather than to imply statistical generalizability. Accordingly, the analysis remains primarily interpretive and grounded in participants’ lived experiences [35, 36].

In the study, participants were first asked when their fear of flying began. Based on the responses provided by the participants:

#### Can you tell me about the first time you remember feeling fear related to flying? What do you think triggered it?

**Fig. 1:**
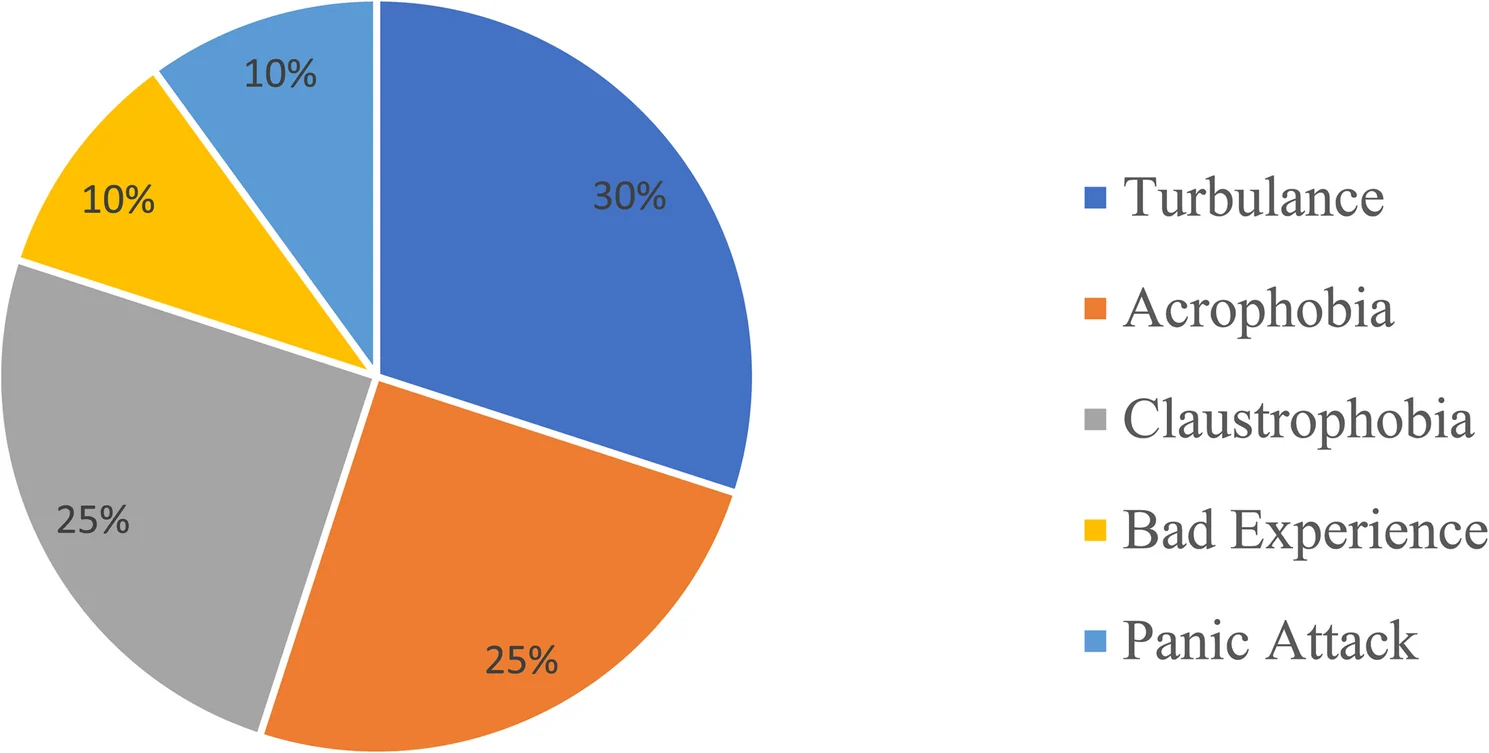
The events that caused fear of flying to start

The stories of how participants’ fear of flying had begun are presented in Fig. 1 and categorized as due to turbulence, which consistently emerged as the most prominent trigger across narratives, as well as acrophobia, claustrophobia, panic attacks without a clear reason, and negative flight experiences. For instance, one participant linked the onset of fear to a specific negative flight experience:

P5: “*It started after negative incidents like when*,* during one of my flights*,* the wing scraped the ground during landing.*”

Similarly, another participant emphasized the role of anticipatory anxiety related to weather conditions:

P18: “*It started the first time I boarded a plane; I remember worrying that if the weather got worse*,* the plane might crash.*”

In addition, indirect exposure to aviation-related content was also identified as a trigger:

P12: “*It started when I was 12*,* on a family holiday. Before the flight*,* I’d watched a documentary about plane crashes*,* and during the flight*,* we encountered turbulence and experienced the things I had seen in the documentary*.”

The story of the fear of flying varies among participants, with the main reason for developing this fear and the duration of the experience also differing.

#### How has your fear of flying changed over time? What keeps it going, in your opinion?

**Fig. 2:**
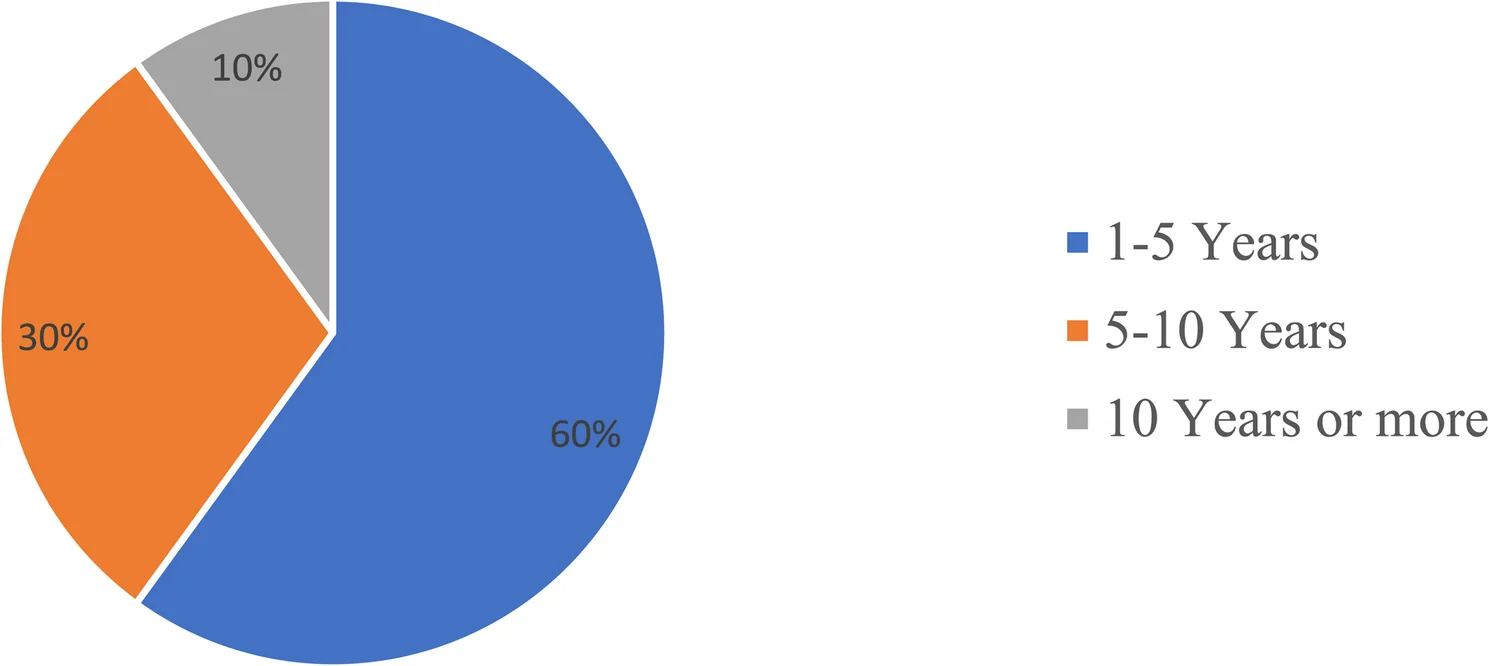
The duration of the fear of flying

Based on participants’ responses, the duration of fear of flying varied (see Fig. 2), with many reporting long-term fear of flying, while others described more recent experiences.

Participants who had experienced fear of flying for extended periods commonly associated the onset of their fear with underlying psychological factors, particularly claustrophobia related to enclosed cabin environments and a sense of suffocation, acrophobia due to high altitude, and panic linked to restricted movement and perceived loss of control.

Participants’ accounts also illustrate the persistence of this fear over time. For example:

P16: “*Since I was a child*,* I think it’s more than 10 years.*”

P9: “*Since the first time I flew*,* almost 16 years ago.*”

These narratives suggest that fear of flying, once developed, may become a long-standing condition, particularly when reinforced by recurring cognitive and emotional responses to flight-related situations.

The turbulence was the reason for those who have had fear of flying for 5 to 10 years and negative flight experience for those less than 5 years.

#### Can you describe situations—before or during a flight—that make your fear worse?

**Fig. 3:**
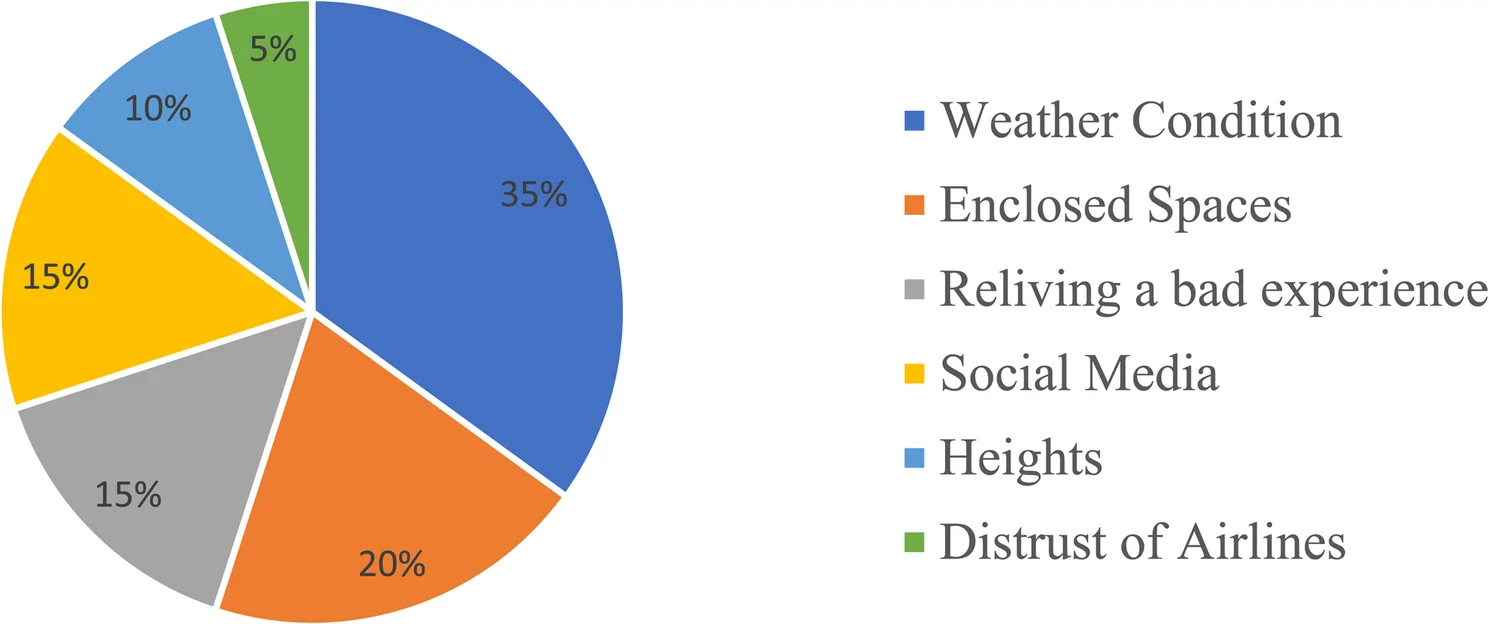
Environmental factors triggering fear of flight

Participants’ responses to this question were classified into several categories (see Fig. 3). Environmental factors, such as weather conditions, emerged as a prominent trigger, followed by enclosed spaces and the fear of reliving negative flight experiences. The influence of news and social media was also frequently mentioned as a factor that intensified fear. In addition, some participants associated their anxiety with heights, while a smaller number referred to a general distrust of airlines.

Participants’ narratives further illustrate how these triggers are experienced in practice. For example, one participant emphasized the role of environmental uncertainty:

P14: “*Weather conditions affect me a lot; I’m afraid of experiencing turbulence because of the weather.*”

Similarly, exposure to aviation-related media content was described as a key source of anxiety:

P9: “*I get very triggered when I see news about plane crashes.*”

And, concerns related to institutional trust and perceived safety standards were also highlighted:

P5: “*Since I’m interested in the industry*,* it affects me because I think airlines don’t do enough in terms of safety.*”

Participants affected by enclosed spaces reported that their fear of flying began when they had to wait for a long time inside the plane due to a delay, and couldn’t disembark. Those affected by meteorological events mentioned that they had previously experienced turbulence and, as a result, had a fear of experiencing it again. Participants influenced by the news indicated that their psychological conditions were associated with the emergence of this fear. Television footage and social media news of events like the September 11 attacks have been linked to PTSD and depression, and it has been found that direct exposure to disaster events has an interactive effect on media consumption [17]. The participants who distrusted airlines closely followed the Boeing 737-Max accidents and mentioned that their fear was reported to be higher following these events.

#### How do stories or news about aviation incidents affect your emotions or decisions about flying?

**Fig. 4:**
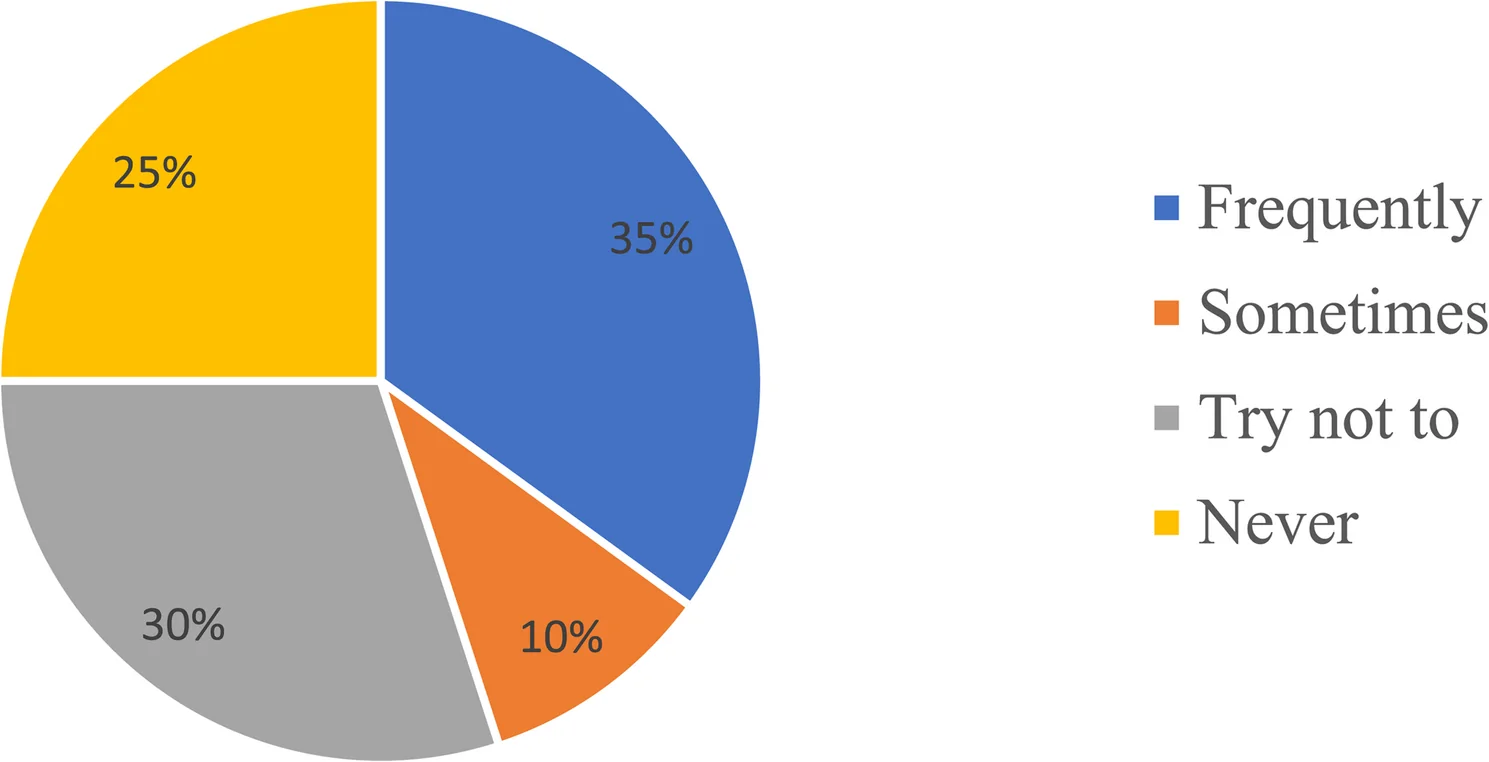
Frequency of following plane crash news

Participants’ engagement with aviation accident news varied (see Fig. 4). Some participants reported that they frequently followed such news, whereas others stated that they deliberately avoided it due to its negative impact on their anxiety. A smaller group indicated occasional exposure, while some reported no engagement at all.

Participants’ statements show that following such news may increase fear, while avoiding it may function as a way to control anxiety. For example, one participant stated:

P20: “*I follow them frequently.*”

In contrast, another participant emphasized avoidance:

P12: “*I try not to follow them too often because it makes me panic more.*”

Similarly, some participants reported a high level of engagement with aviation-related incidents:

P17: “I follow them very closely; I know about every plane crash.”

It has been observed that the group that does not particularly follow plane crash news is negatively affected by these reports and tends to panic. Individuals in this group with a fear of flying have stated that they change the channel when they come across such news.

#### Where do you usually hear about aviation-related accidents? How do these sources influence your thoughts about flying?

**Fig. 5:**
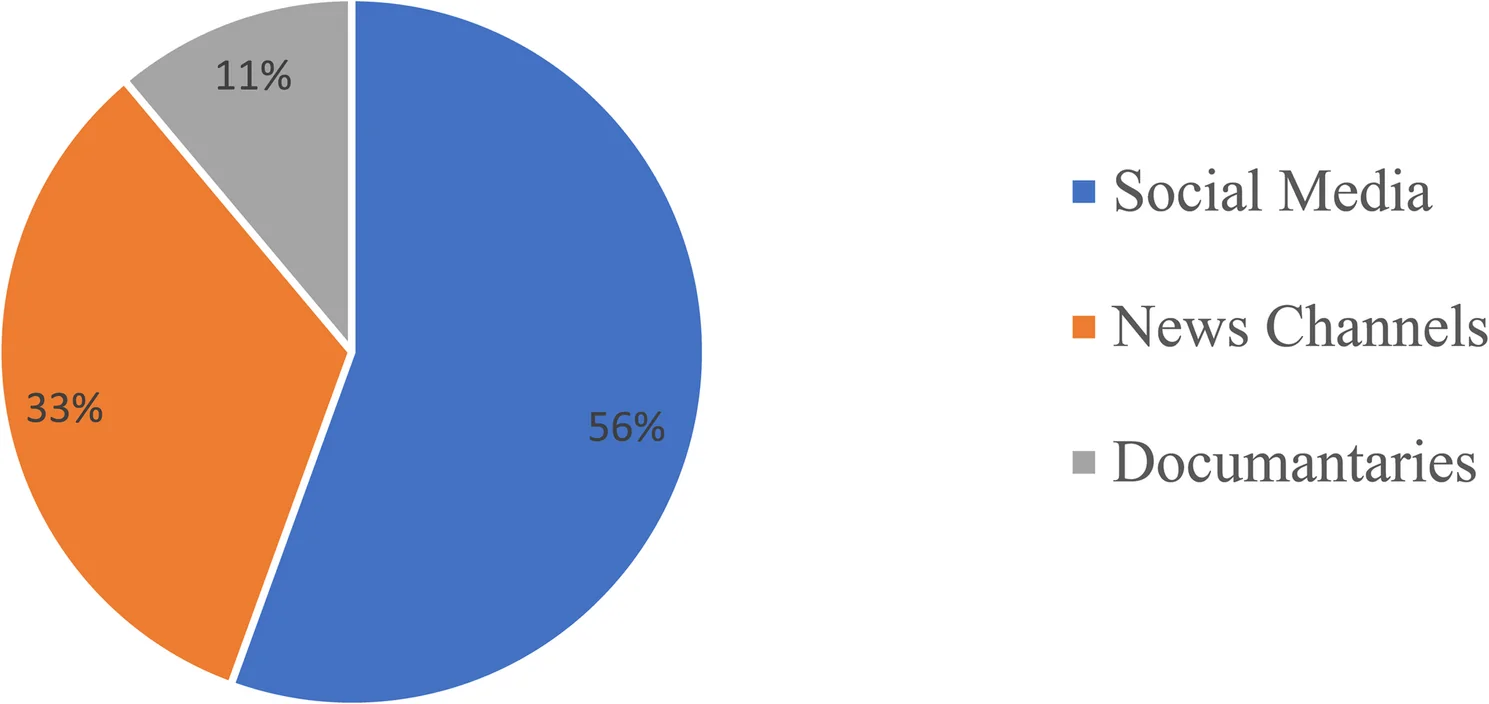
News sources

Most participants reported that they follow news about airplane crashes primarily through social media (see Fig. 5), which is a platform open to various forms of manipulation [42]. Others indicated that they follow such news through traditional news channels, while a smaller number referred to documentaries. Participants’ statements also support this pattern. For example:

P10: “*I follow them through social media.*”

Similarly, another participant stated:

P9: “*I follow them through news channels.*”

This suggests that social media plays a central role in shaping exposure to aviation-related information.

#### Can you walk me through your emotional state during the time leading up to a flight? What thoughts or feelings do you experience?

**Fig. 6:**
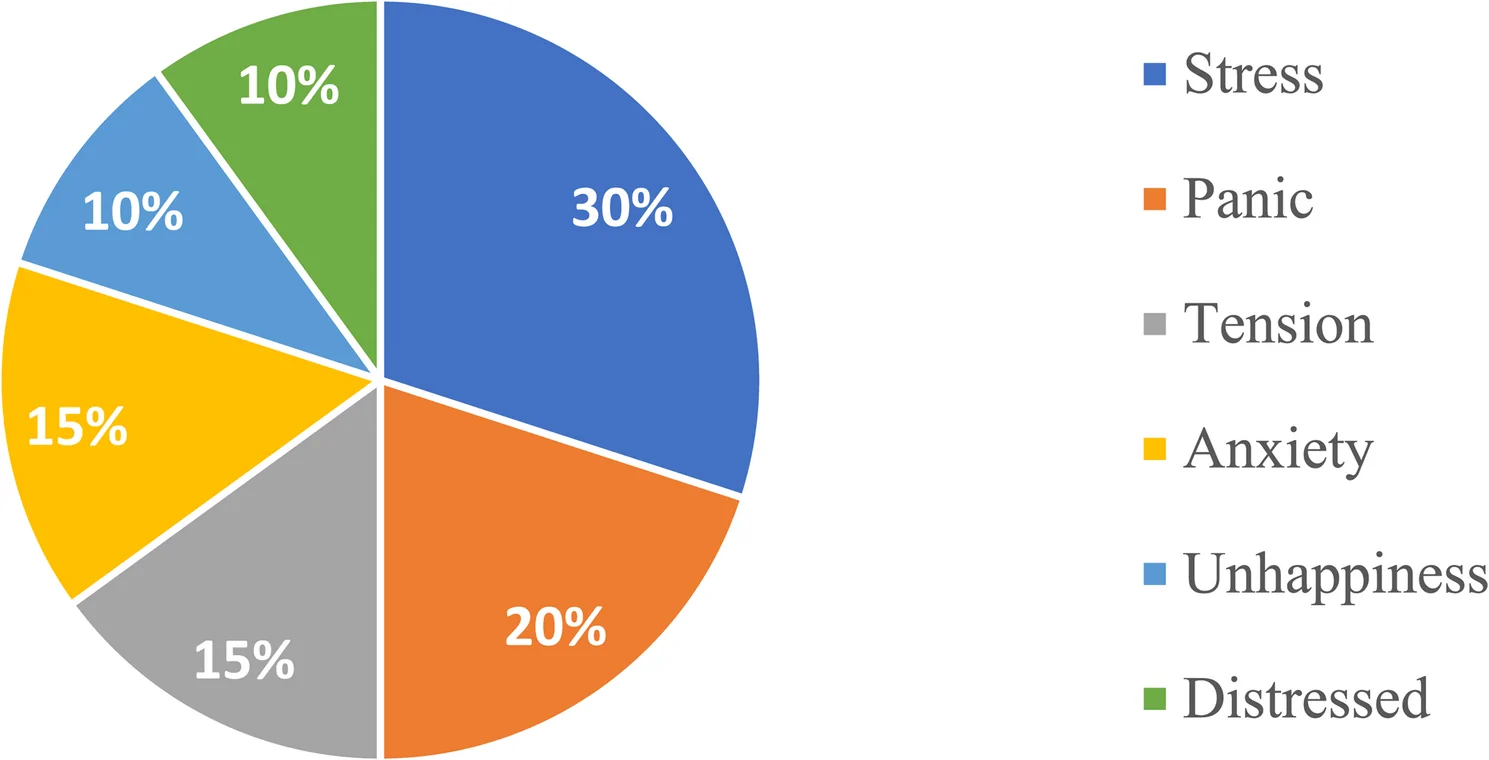
Feelings during the preparation for the flight

Participants reported experiencing different emotional states during the preparation process (see Fig. 6). Stress, anxiety, and panic were the most commonly expressed emotions, while tension, unhappiness, and general distress were also mentioned by some participants.

Participants’ statements indicate that these emotions may begin before the flight and intensify as the departure approaches. For example, one participant stated:

P13: “*I feel a bit anxious; the thought of turbulence is frightening and triggering.*”

Similarly, another participant described how anticipatory thoughts lead to panic:

P5: “*While packing*,* I think about boarding the plane and start to panic*,* imagining that I will feel uncomfortable during the flight.*”

In addition, some participants reported that anxiety builds progressively across different stages of the travel process:

P19: “*My anxiety starts weeks before the flight. On the way to the airport*,* when I arrive*,* while boarding*,* and once I’m on the plane*,* my anxiety intensifies at every stage.*”

Generally, participants who had fear of flying for 10 years or more experienced stress before the flight. Participants who were experiencing panic were also experiencing distress at the same time. Participants who had previous negative flight experiences had anxiety and tension, and those with acrophobia and claustrophobia had emotions of unhappiness and distress.

#### During the flight, what kinds of thoughts or emotions come up for you? Can you describe a recent experience?

**Fig. 7:**
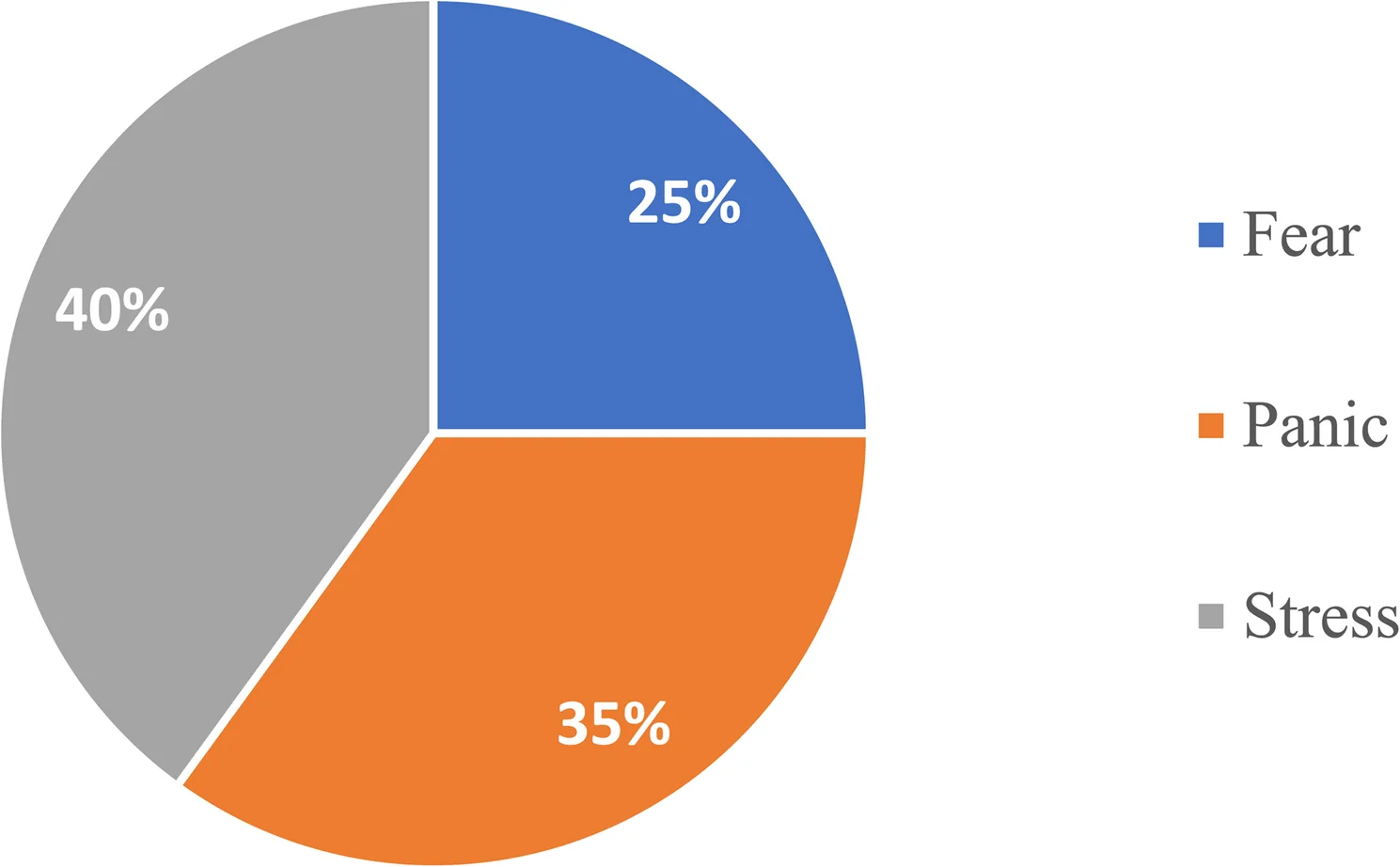
Feeling during the flight

As shown in Fig. 7, participants reported experiencing fear, panic, and stress during the flight. Among these, stress appeared as the most prominent emotional response, followed by panic and fear.

Participants’ statements illustrate these experiences. For example, one participant stated:

P12: “*As soon as the flight begins*,* I start to feel afraid.*”

Similarly, another participant described experiencing intense panic:

P2: “*I go into a severe panic; I feel terrible and can’t focus on anything.*”

In addition, some participants emphasized the level of stress experienced during the flight:

P16: “*I experience great stress.*”

These findings suggest that emotional responses during flight are not limited to a single form of anxiety but involve overlapping experiences of fear, panic, and stress. Participants who reported panic often also referred to enclosed cabin environments and turbulence as contributing factors. Moreover, higher levels of stress and fear were more commonly expressed by individuals with prior negative flight experiences or underlying psychological conditions.

#### What kinds of physical sensations do you notice when you’re afraid during a flight? What do you think they mean to you?

Some situations can trigger the release of stress hormones, leading to physical reactions such as increased heart rate, rapid breathing, and sweating. These responses are part of the “fight or flight” mechanism [43] and may occur in passengers who perceive flying as a threat.

**Fig. 8:**
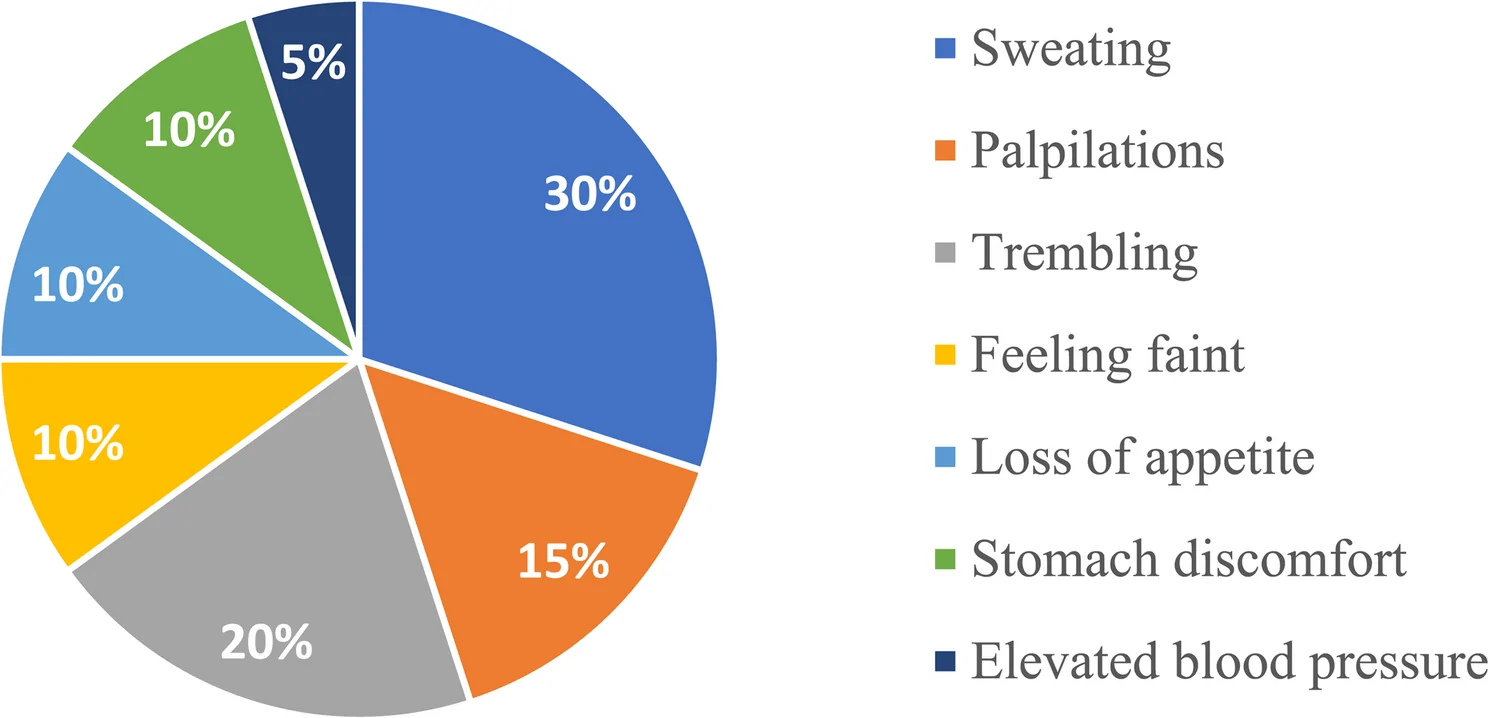
The symptoms that experienced during the flight?

Participants reported experiencing a range of physical symptoms during flights (see Fig. 8). Sweating emerged as the most prominent response, followed by trembling, palpitations, and feelings of faintness. In addition, some participants mentioned stomach discomfort, loss of appetite, and increased blood pressure.

Participants’ statements illustrate these experiences. For example:

P8: “*During the flight*,* I get very anxious and start sweating.*”

Similarly, another participant described more intense physiological reactions:

P19: “*I experience symptoms like nausea and a feeling of fainting—similar to what the body goes through when expecting terrible news.*”

In addition, increased heart rate was also emphasized:

P10: “*My heart rate increases significantly*,* and I start sweating a lot.*”

These findings indicate that physical symptoms during flight are diverse but closely related to anxiety responses, often occurring simultaneously and reinforcing the overall experience of fear.

#### Are there particular parts of the flight that are more difficult than others (e.g., takeoff, turbulence)? What makes them harder for you?

**Fig. 9:**
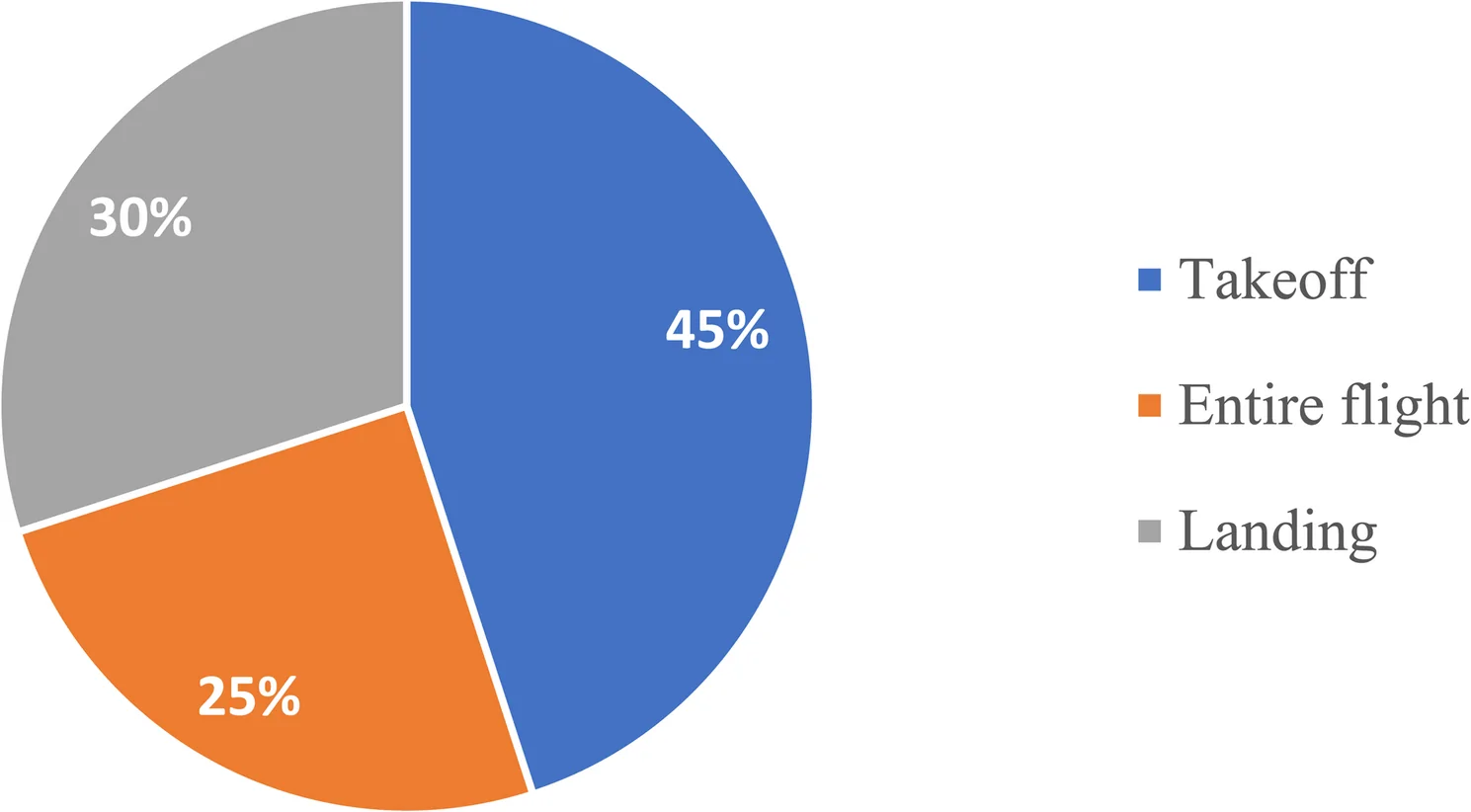
The situations increase the intensity of the fear of flying

Participants indicated that their fear intensifies during specific phases of the flight, particularly during takeoff, landing, or throughout the entire flight, often triggered by even minor signs of turbulence (see Fig. 9). Fear was most commonly associated with takeoff, followed by landing, while some participants reported persistent fear throughout the entire flight.

Participants’ statements illustrate these patterns. For example:

P11: “*The intensity increases the moment I sit in my seat on the plane*,* and the entire flight becomes very difficult.*”

Similarly, another participant emphasized the impact of aircraft movement during takeoff:

P13: “*The intensity of my fear increases during takeoff because the plane shakes.*”

In line with previous findings, participants who reported heightened fear during takeoff often had a history of negative flight experiences and long-term fear of flying. A common factor among those experiencing increased fear during takeoff and landing was discomfort with aircraft movement. Participants who reported fear throughout the entire flight frequently referred to claustrophobia and a continuous sense of unease from boarding to landing. In contrast, those who experienced heightened fear during landing often associated their anxiety with prior exposure to aviation accidents and related media content.

#### What do you do to help yourself feel calmer or safer during a flight? How effective are those methods for you?

**Fig. 10:**
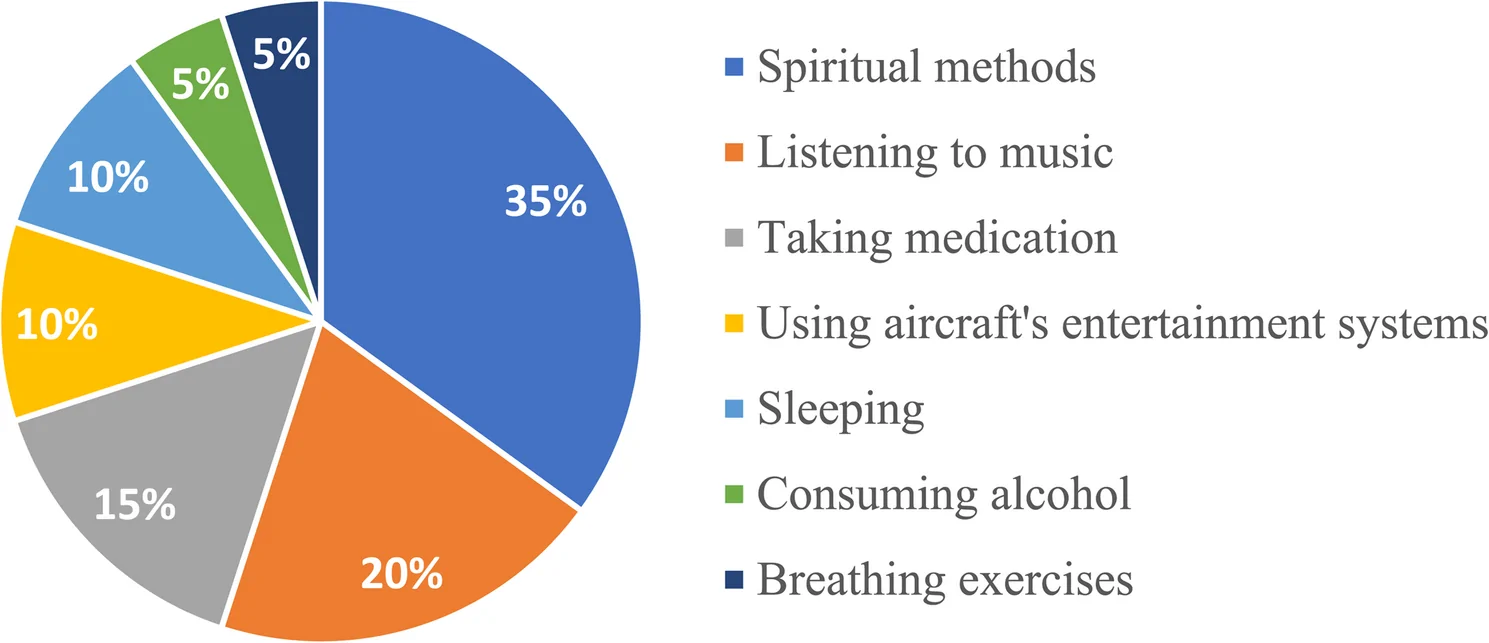
Adopted relaxation methods during the flight

When participants were asked about the methods they used to relax during the flight, a variety of coping strategies emerged (see Fig. 10). Participants often reported using more than one method simultaneously, depending on the intensity of their anxiety and the stage of the flight.

Spiritual practices, such as praying or thinking positively, were among the most commonly mentioned strategies. In addition, participants referred to listening to music, taking medication before the flight, and using in-flight entertainment systems as ways to distract themselves. Some participants also reported trying to sleep, controlling their breathing, or, in some cases, consuming alcohol to manage their anxiety.

Participants’ statements reflect these coping patterns. For example:

P4: “*Spiritual practices help me. I pray and think that everything will be fine.*”

Similarly, another participant emphasized the use of medication:

P10: “*I usually take calming medication.*”

In addition, distraction-based strategies were also highlighted:

P20: “*I usually focus on external factors around me*,* such as the in-flight entertainment system and the internet.*”

Participants who relied on methods such as sleeping, breathing control, or alcohol often associated their fear with prior negative flight experiences, such as turbulence or rough landings. Furthermore, some participants indicated that they depend on social support during flights, stating that they feel more comfortable when accompanied by others and may struggle to fly alone.

#### What are the most distressing thoughts or images that enter your mind when you’re on a plane?

**Fig. 11:**
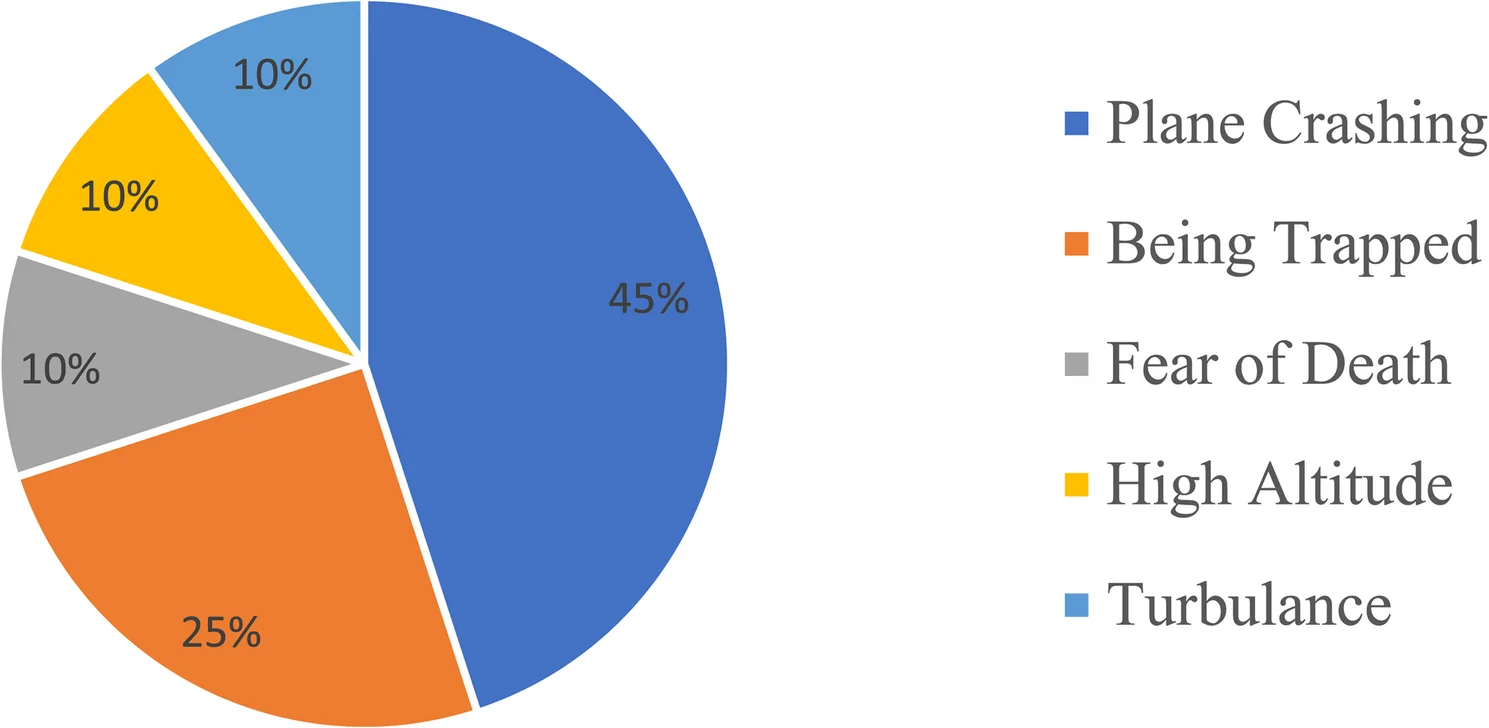
Bothering thoughts during the flight

Participants were asked about the thoughts that disturbed them during the flight, and several recurring themes emerged (see Fig. 11). The most prominent concern was the possibility of a plane crash, followed by the fear of being trapped inside the aircraft for an extended period. Other disturbing thoughts included fear of death, discomfort related to high altitude, and concerns about turbulence.

Participants’ statements reflect these patterns. For example, one participant expressed fear of death linked to a potential accident:

P5: “I experience a fear of death, thinking that if something goes wrong, I won’t make it out of the plane alive.”

Similarly, another participant emphasized the role of turbulence in triggering discomfort:

P13: “The thought that the plane might encounter turbulence makes me uncomfortable.”

In addition, concerns related to being confined for a prolonged period were also reported:

P3: “If the flight gets delayed and I have to stay on board for a long time,”

These findings suggest that disturbing thoughts during flight are primarily centered around loss of control, perceived danger, and confinement, often reinforced by prior experiences and exposure to aviation-related information.

#### How has your fear of flying influenced your life—your travel, your work, or your relationships?

**Fig. 12:**
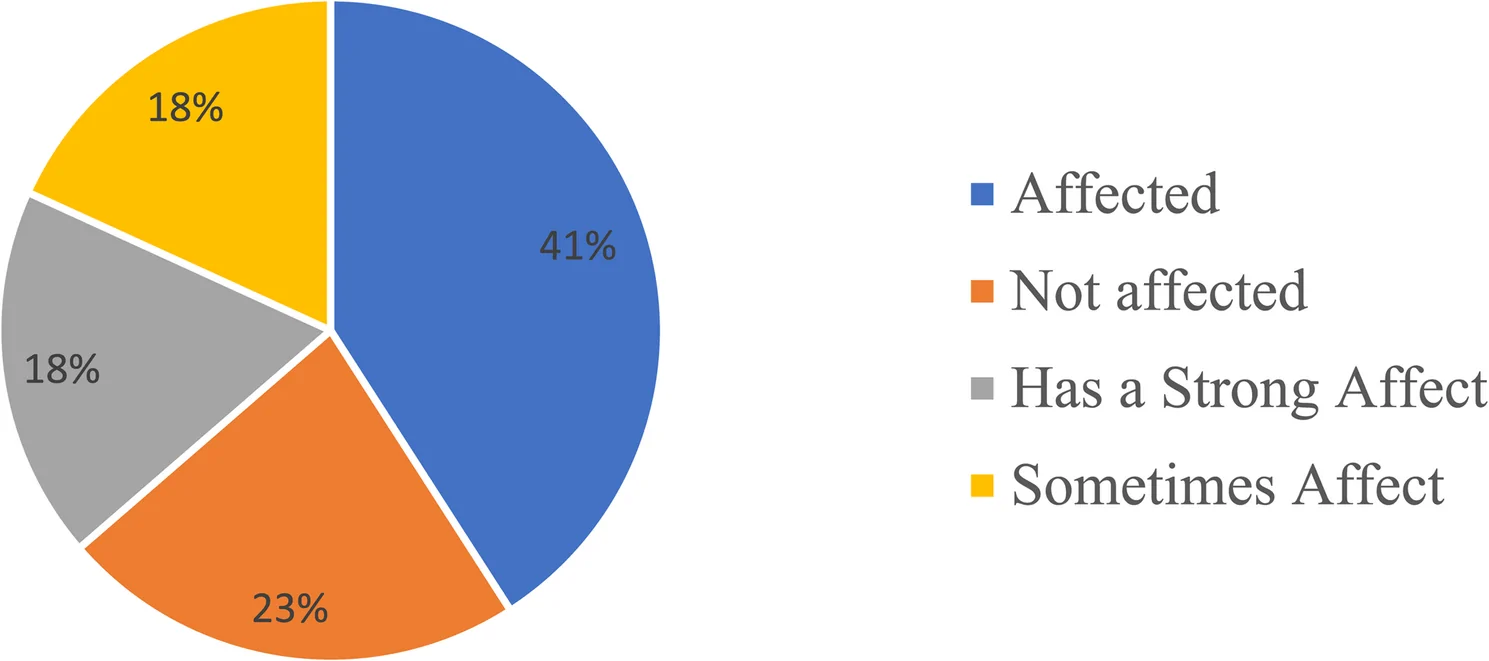
How phobias affect travel plans

Fear of flying was found to impact participants’ lives socially, psychologically, and in terms of comfort. When asked whether their fear of flying affects their travel plans, participants reported varying levels of influence (see Fig. 12). For some, fear of flying directly limited their mobility, leading them to avoid air travel and choose alternative modes of transportation despite the disadvantages in time and comfort. Others indicated that they continued to fly despite their fear, often experiencing discomfort but doing so to avoid further restriction in their lives. In addition, some participants reported that the impact of fear of flying depended on situational factors such as travel distance or necessity.

Participants’ statements illustrate these differences. For example, one participant emphasized the restrictive impact of fear of flying:

P6: “*It especially affects my travel plans; unfortunately*,* I can’t go anywhere.*”

In contrast, another participant described a more conditional approach to travel decisions:

P13: “*It doesn’t affect me much if I’m going somewhere nearby—I go by car. If it’s a long distance*,* I get on the plane*,* though it’s difficult for me.*”

These findings suggest that fear of flying does not uniformly lead to avoidance but instead shapes travel behavior along a spectrum, ranging from complete avoidance to reluctant engagement, depending on contextual factors.

#### Have you ever considered or tried any support (therapy, medication, airline programs)? What influenced that decision?

**Fig. 13:**
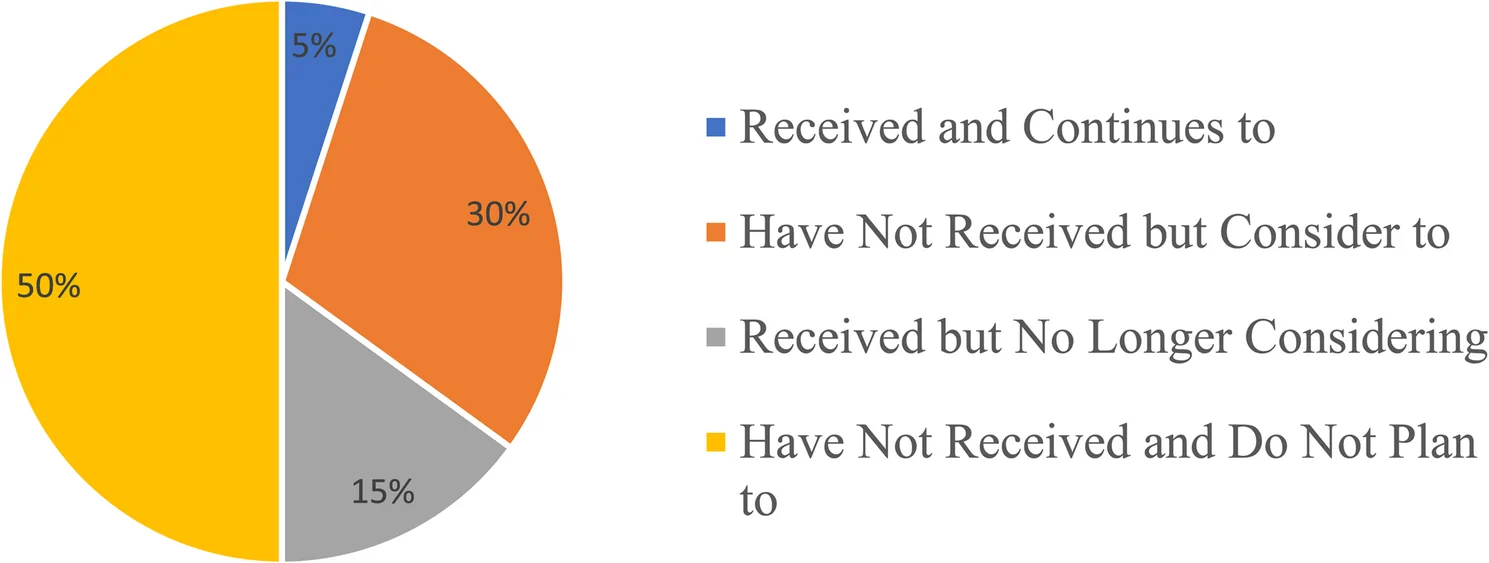
Proffesional support

The final question asked participants whether they had sought professional support to overcome their fear of flying. Participants reported different approaches to seeking support (see Fig. 13). While a small number had previously received professional help, most participants had not sought support. Among those who had not received support, some expressed willingness to consider it in the future, whereas others indicated that they did not plan to seek professional help. Reasons for not seeking support included believing it would be ineffective, rarely flying, or preferring to manage the fear independently.

Participants’ statements reflect these differences. For example, one participant expressed openness to seeking support due to the impact of fear of flying on daily life:

P19: “*I haven’t received it*,* but I’m considering it*,* because I would rather not lose the dozens of hours I’ve lost by choosing trains or especially buses due to my fear.*”

In contrast, another participant preferred to cope without professional help:

P4: “*I haven’t received it. I hope to overcome it on my own.*”

In addition, some participants reported prior engagement with professional support:

P10: “*Yes*,* I have received professional support before; I had a few sessions with a psychologist.*”

These findings suggest that help-seeking behavior in fear of flying is limited and varies across individuals, often shaped by personal beliefs, perceived necessity, and previous experiences with fear.

### Analyzing the data

Thematic analysis was conducted using MAXQDA 2024, following the six-phase approach outlined by [44]: (1) familiarization with data, (2) generating initial codes, (3) searching for themes, (4) reviewing themes, (5) defining and naming themes, and (6) writing up. An inductive coding strategy was employed to allow themes to emerge organically from the data, without imposing preexisting categories.

Two researchers independently coded the transcripts using MAXQDA’s “Code System” feature. Initial open coding was followed by axial coding to refine thematic categories and explore interconnections between psychological, behavioral, and contextual elements of fear of flying. MAXQDA’s “Code Relations Browser” and “Code Co-occurrence Model” were used to analyze the frequency and strength of associations between codes (e.g., ‘turbulence’ × ‘panic’). This allowed us to visually and quantitatively explore how symptoms, triggers, and coping strategies clustered across participant narratives. It should be noted that code co-occurrence analysis was used to identify patterns of association between themes within participants’ narratives. These patterns are interpreted as relational rather than causal, reflecting the co-presence of experiences rather than directional or explanatory relationships.

Disagreements during coding were discussed collaboratively until consensus was reached. To enhance trustworthiness and credibility, intercoder reliability was assessed using MAXQDA’s interrater agreement tool (κ = 0.84), indicating strong agreement. A third expert was consulted to resolve remaining discrepancies in code definitions. Reflexive memos were maintained throughout the analysis process to capture emerging ideas, potential biases, and methodological decisions. These memos were reviewed weekly during team meetings to promote analytic transparency and methodological rigor.

In addition, MAXQDA’s visual tools (e.g., word clouds and document portraits) were used to support data interpretation and triangulate findings. The single-case model was created by aggregating code co-occurrence frequencies across all participants to identify shared experiential patterns of fear of flying.

Reflexivity was strengthened by explicitly considering the researcher’s positionality throughout the study. The research team, with academic backgrounds in aviation management, may hold pre-existing assumptions regarding aviation safety and passenger behavior, which could introduce interpretive bias. To address this, ongoing critical reflection and regular team discussions were used to examine assumptions and ensure that interpretations remained grounded in participants’ narratives.

**Fig. 14:**
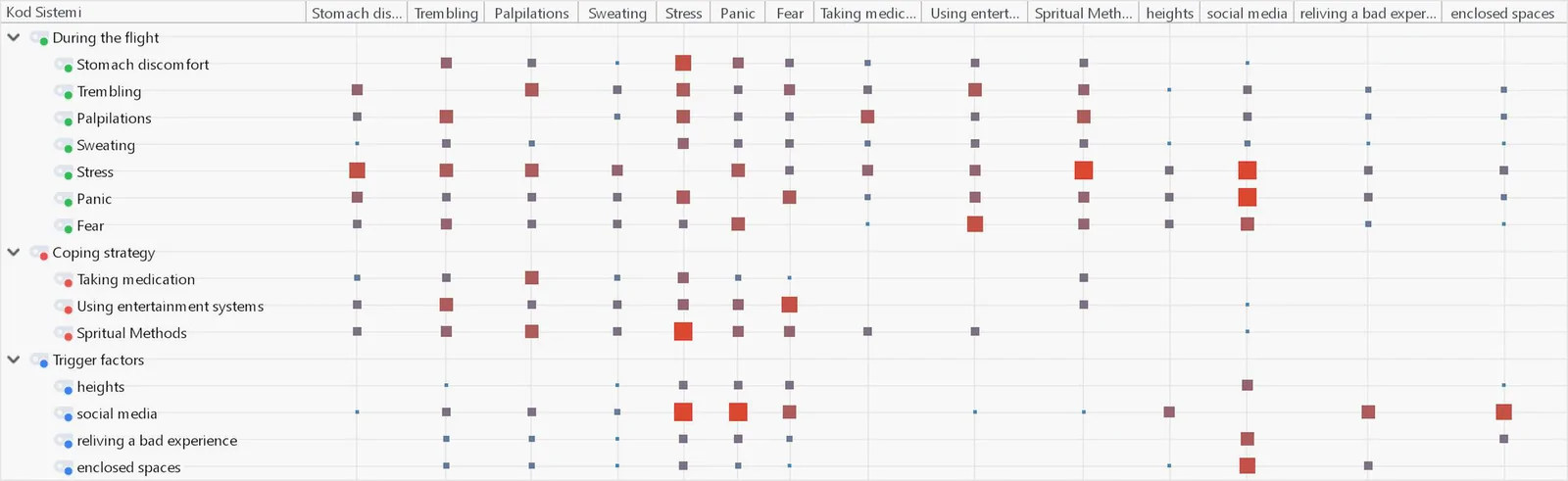
Code relationships

The frequency with which the codes (Fig. 14) were coded together in a single sentence or paragraph was used to investigate the links between them. This investigation led to an evaluation of the relationships between different codes. This assessment’s main objective was to identify the codes’ strong and weak links. The components utilized in the participants’ assessments were also taken into account.

The findings revealed the following key relationships:

- The social media code, considered a triggering factor, was used 18 times in conjunction with stress and 17 times with panic. This relationship highlights the association between negative flight-related content on social media and passenger responses. The social media code was also associated 14 times with the fear of enclosed spaces. Frequent sharing of narrow cabin images, turbulence videos, or negative flight experiences on social media may be associated with increased anxiety of individuals with claustrophobia. Such content is described by participants as increasing feelings of confinement and insecurity during flights. As a result, passengers with claustrophobia may consciously or unconsciously reinforce their fears through the content they see on social media.
- In terms of coping strategies, spiritual methods such as praying or thinking positively are strongly associated with symptoms like stress (18 times) and panic (10 times). This suggests that spiritual methods are often preferred as effective coping mechanisms in situations of intense stress. Additionally, code relationships indicate that fearful passengers prefer listening to music or watching movies (14 times), while those experiencing heart palpitations tend to take medication before flights.
- Regarding emotional and physical symptoms, the stress code was associated with the stomach discomfort code 14 times. This relationship may reflect the association between stress and digestive discomfort during flights. The stress code was also associated with the panic code (13 times), which indicates that feelings of panic and stress frequently co-occur, and the two conditions frequently interact with each other.

As per the results, social media platforms are strongly associated with increased levels of fear of flying, particularly in the context of exposure to aviation-related negative news and content, such as accidents, terrorist attacks, or flight cancellations. This finding is in line with the research suggesting that excessive exposure to aviation-related negative news on social media can lead to an exaggerated perception of risk, influencing public sentiment and increasing travel-related anxieties [45]. Furthermore, social media often highlights predominantly catastrophic events, leading to increased public anxiety and avoidance behaviors [23, 46]. As a result, some individuals may begin to associate air travel with danger, which may be associated with avoidance behaviors.

Social media and news effects were particularly evident following a series of major aviation accidents in late 2024. During this period, over 300 fatalities were reported across multiple commercial and military aircraft crashes worldwide. The accidents were primarily attributed to technical failures, adverse weather conditions, and operational errors during critical flight phases such as takeoff and landing. A follow-up study was conducted with the 12 participants to assess the psychological impact of the recent aviation accidents. All participants reported that the recent news of aircraft accidents in Azerbaijan, South Korea, and the USA was associated with increased fear of flying. Furthermore, 10 participants indicated the likelihood of canceling their upcoming flights, while two expressed the intention to conduct further research on the accidents before deciding whether to proceed with their travel plans.

The follow-up study findings highlight social media’s dual impact on individuals’ perceptions of air travel. While most of the participants reported increased anxiety and avoidance behaviors due to distressing news, others attempted to mitigate their fear of flying through information gathering, relying on more in-depth analyses to form their judgments. This finding is also in line with the research conducted by [22], which revealed that some individuals succumb to fear-based narratives while others engage in fact-checking and data-driven assessments to counteract misinformation.

**Fig. 15:**
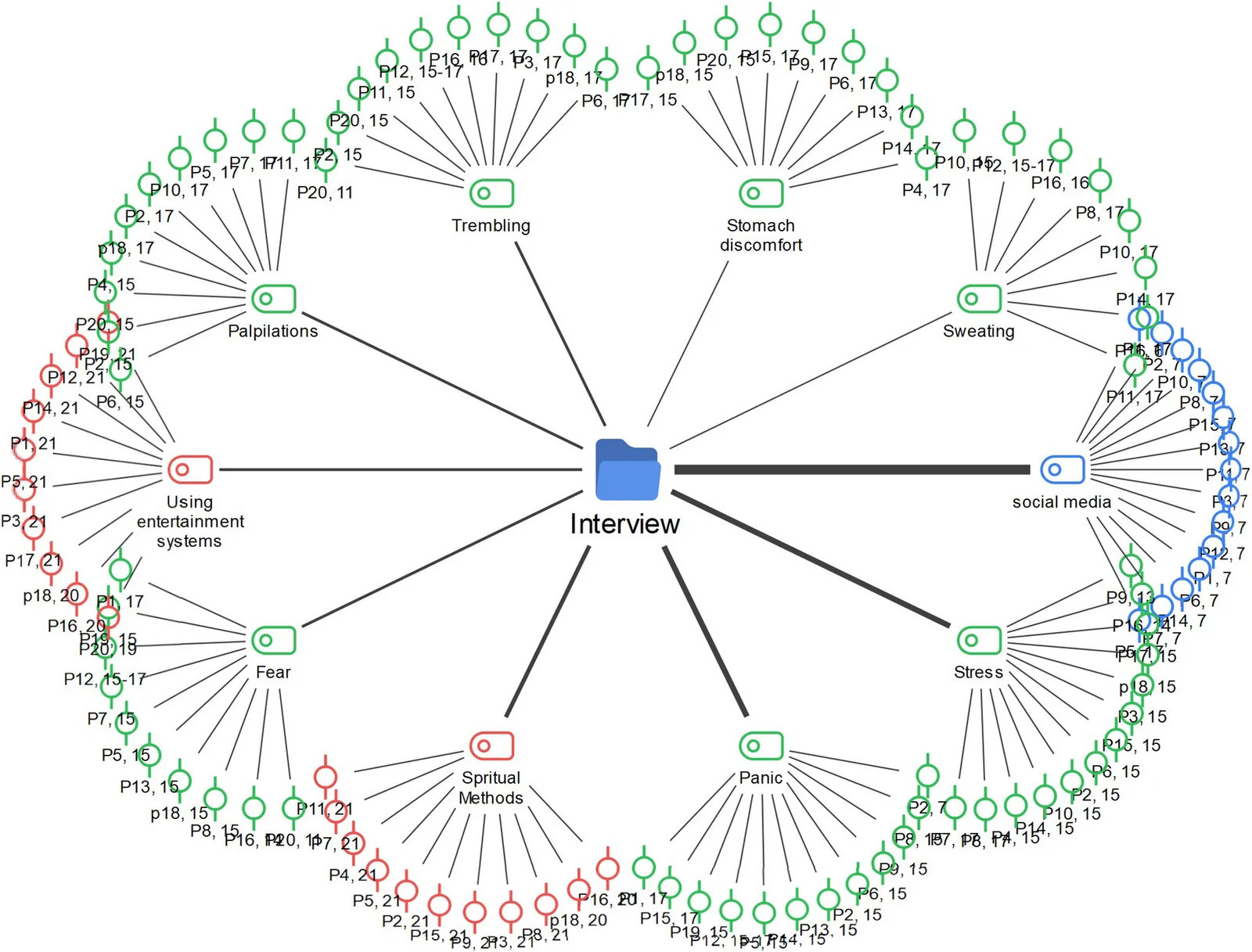
Single case model

We also conducted the *single-case model analysis*, which treats the responses of all participants as if they were provided by a single individual. During this process, participants’ responses are combined to form a unified data set represented by a single integrated code relationship. The single-case model analysis aims to identify the shared concerns and common perspectives of participants. The Single-Case Model is given in Fig. 15. The thickness of the line indicates the frequency of code usage, and the single-case model progresses clockwise, starting from the thickest line. The single case model showed that the shared concerns revolved around the role of social media, stress factors, and feelings of panic.

**Fig. 16:**
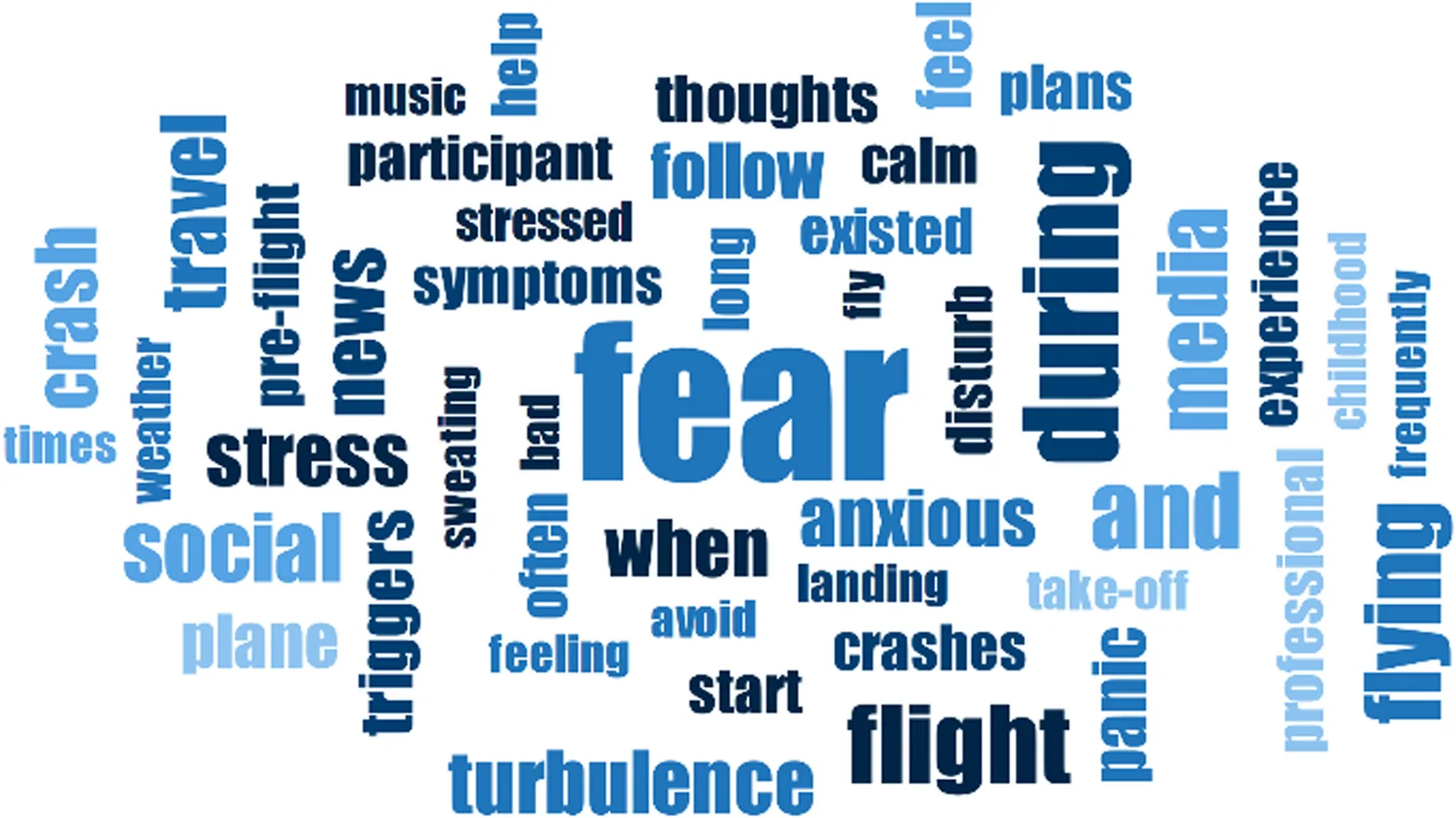
Word cloud

The word cloud (Fig. 16) developed through the MAXQDA program provides a further interpretation of participants’ responses. The sizes of the words in the visualization indicate the significance of factors triggering levels of fear. Besides the most frequently used word, “fear” terms such as “turbulence,” “news,” and “crash” also stand out prominently.

## Discussion

This study explored key psychological, environmental, and behavioral factors associated with fear of flying among participants from Türkiye. Thematic analysis revealed six dominant categories: psychological factors (e.g., claustrophobia, anxiety), negative flight-related experiences (e.g., turbulence or crash exposure), cognitive distortions, social influences, physiological symptoms, and culturally shaped beliefs. These categories align with, and in some cases expand upon, previous studies conducted in Western contexts [2, 14, 47]. Taken together, these findings suggest that fear of flying should not be interpreted as a single-factor phenomenon, but rather as a multidimensional construct shaped by the interaction of individual vulnerabilities (e.g., anxiety sensitivity), environmental exposures (e.g., media), and sociocultural meaning systems. This integrative perspective is also consistent with contemporary models of anxiety, which emphasize the interaction between cognitive appraisal processes and contextual triggers [48]. Building on this multidimensional framework, the following discussion elaborates on how these factors interact in shaping the emergence, maintenance, and expression of fear of flying.

Turbulence and enclosed spaces were consistently cited as core triggers, mirroring previous quantitative findings that link turbulence-related fear with anticipatory anxiety and panic disorders [5, 13]. However, this study suggests how cultural context (e.g., reliance on spiritual coping mechanisms) and media saturation (especially through social media) may be associated with increased fear levels. This supports recent work on the emotional contagion effects of digital platforms [22, 46]. More importantly, these findings point toward a central underlying mechanism—perceived loss of control—which has been widely identified as a core feature of anxiety disorders [49]. Participants’ heightened sensitivity to turbulence and confined spaces appears to reflect not only situational discomfort but also a deeper cognitive schema related to unpredictability and helplessness. This aligns with cognitive theories suggesting that individuals with anxiety disorders tend to interpret ambiguous stimuli as threatening [19], thereby amplifying otherwise normative flight experiences into perceived danger. In this sense, situational triggers such as turbulence should not be understood in isolation, but rather as activating pre-existing cognitive and emotional vulnerabilities.

Social media emerged as a commonly reported trigger of fear of flying, particularly among participants with underlying anxiety. Many described avoiding flights after encountering crash videos or negative news online. This pattern is consistent with the findings of Cheng, Mitomo, Otsuka, and Jeon [23], who reported that repeated exposure to post-disaster media content is associated with heightened risk perception and behavioral responses. From a theoretical standpoint, this finding can be further interpreted through the lens of the availability heuristic, where individuals estimate the likelihood of events based on how easily examples come to mind. Frequent exposure to aviation accidents in digital media may therefore distort risk perception, leading individuals to overestimate the probability of rare events. Additionally, cultivation theory [50] suggests that long-term exposure to mediated content can shape individuals’ perceptions of reality, which may explain why participants reported persistent anxiety despite objective evidence of aviation safety.

All these mechanisms indicate that media exposure does not merely inform individuals, but actively reshapes their cognitive representations of risk.

Participants also described various coping strategies: spiritual rituals (praying), distraction (music, movies), medication, and avoidance. Although CBT and exposure therapy are clinically validated [26, 29], most participants reported never seeking professional help—due to stigma, lack of access, or fatalistic beliefs. This finding aligns with van [7], who observed similar underutilization in European samples. It further suggests a need for culturally tailored interventions in countries like Türkiye. At this point, the findings highlight a critical gap between the availability of evidence-based treatments and their actual utilization, suggesting that psychological accessibility and cultural compatibility may be as important as clinical effectiveness. In particular, the prominence of spiritual coping strategies indicates that individuals may prioritize culturally familiar and emotionally meaningful approaches over formal therapeutic methods. This discrepancy further reinforces the importance of contextualizing intervention strategies within the sociocultural environment in which fear is experienced.

Importantly, the findings also indicate that avoidance behavior in fear of flying is not always absolute but may be shaped by situational constraints. Several participants reported that they actively avoid flying whenever possible, yet are occasionally compelled to fly due to external obligations such as family decisions or professional requirements. For instance, one participant working in an airline technical department stated: “I never fly unless I absolutely have to.” This statement illustrates that engagement in air travel does not necessarily reflect the absence of fear, but rather the presence of contextual pressures that override avoidance tendencies. In line with this, previous research suggests that individuals with specific phobias may endure the feared situation when avoidance is not feasible, while still experiencing significant distress [1]. Therefore, fear of flying should be understood not only through observable behavior (i.e., flying vs. avoidance), but also through the subjective experience of fear and perceived lack of control. This distinction is particularly critical for interpreting qualitative findings, where subjective meaning-making takes precedence over purely behavioral classifications.

A particularly novel contribution is the use of MAXQDA’s code co-occurrence and single-case model analysis, which revealed strong interaction patterns between specific fear triggers (e.g., media exposure and claustrophobia) and symptom profiles (e.g., panic and stomach discomfort). These empirical linkages provide insight into the patterns of fear of flying, consistent with a biopsychosocial framework for understanding flight anxiety. These patterns further demonstrate that fear of flying is not randomly structured but follows identifiable relational dynamics, where certain triggers systematically co-occur with specific emotional and physiological responses. This strengthens the argument that fear of flying should be understood through an integrated framework that captures the dynamic interplay between cognitive, emotional, and contextual factors.

Accordingly, the findings move beyond descriptive categorization and contribute to a more systemic understanding of fear of flying as an interconnected network of experiences, perceptions, and responses.

## Conclusion

This study explored the psychological and behavioral mechanisms underlying fear of flying among Turkish air travelers, contributing region-specific insight to a field dominated by Western samples.

The findings are consistent with previous research and suggest that fear of flying is multifactorial, involving not only internal vulnerabilities (e.g., anxiety, negative memories), but also external influences such as digital media, misinformation, and cultural values. The study also highlights a gap between fear severity and professional help-seeking, indicating potential areas for public education, early intervention, and airline-based support programs.

These insights hold significance for clinical psychologists, aviation health professionals, and airline policy makers. Incorporating virtual reality exposure therapy (VRET), CBT-based psychoeducation, and spiritual sensitivity into treatment protocols may improve accessibility and acceptance.

Moreover, the findings highlight the need for interventions that counteract aviation-related misinformation on social platforms. Awareness campaigns by airlines and regulatory agencies could mitigate anxiety and correct risk misperceptions.

Future research should build on these findings by conducting longitudinal or mixed-method studies to explore causal relationships between media exposure and severity of fear of flying, and to test the effectiveness of culturally adapted intervention programs.

## Implications for practice

The findings of this study have several practical implications for mental health professionals, airlines, and individuals who have fear of flying. Cognitive-behavioral therapy (CBT) and exposure therapy, particularly virtual reality exposure therapy (VRET), are widely used interventions for reducing flight anxiety. Mental health practitioners should consider incorporating these evidence-based approaches into their treatment plans. Additionally, relaxation techniques, such as deep breathing and mindfulness meditation, can be promoted as accessible coping strategies for individuals experiencing flight-related anxiety.

Airlines and aviation authorities can play a proactive role in addressing fear of flying by offering educational programs that demystify aviation safety and provide practical coping tools. Programs like Turkish Airlines’ “Overcoming Fear of Flying Program” and Lufthansa’s seminars demonstrate the potential of airline-sponsored initiatives to alleviate fear and build passenger confidence. Furthermore, the study underscores the need for greater awareness and accessibility of professional support, as many participants reported underutilization of available resources. Public health campaigns and online self-help tools could bridge this gap, offering individuals the means to manage their fear independently.

## Limitations

While this study provides valuable insights into fear of flying, it is not without limitations. First, the sample size of 20 participants, though sufficient for qualitative research, may limit the generalizability of the findings. Future studies could expand the sample size and include participants from diverse cultural and geographical backgrounds to enhance the external validity and comparison of the results.

Additionally, the study focused primarily on participants from Türkiye, which may limit the applicability of the findings to other cultural contexts. Cross-cultural studies are needed to explore how cultural beliefs and practices influence the experience and management of fear of flying. Finally, the study’s qualitative design, while rich in depth, does not allow for causal inferences. Longitudinal or experimental studies could further elucidate the causal relationships between fear of flying and its contributing factors.

Although efforts were made to limit clustering by restricting referral chains, the use of snowball sampling may still have introduced selection bias by over-representing socially connected individuals.

In addition, as is typical of snowball sampling designs, formal refusal or response rates were not systematically recorded. Unlike probability-based survey designs where researchers directly approach a predefined sampling frame and can document contact attempts and refusals, snowball sampling relies on participant-driven recruitment through informal referral chains, making it methodologically inappropriate to calculate conventional response metrics [51]. Willingness to participate tends to be higher in such designs due to existing trust relations between recruiters and recruits.

Furthermore, the study relied on self-reported fear of flying rather than clinically diagnosed fear of flying, which may limit the distinction between subclinical fear and formally defined specific phobia. While this approach is consistent with the exploratory nature of qualitative research, it should be considered when interpreting the findings.

In addition, although participants were required to have flown within the last year to improve recall accuracy, their narratives may still be subject to recall bias. Social desirability bias may also have influenced how participants described their emotions and coping strategies.

Finally, the study adopts a spectrum-based conceptualization of fear of flying, which is appropriate for capturing a range of lived experiences but may differ from strictly clinical classifications based on formal diagnostic criteria. Therefore, the findings should be interpreted within the context of subjective fear experiences rather than clinical diagnosis.

## Data Availability

Data is available upon request.

## Abbreviations

CBT: Cognitive Behavioral Therapy
DSM-5-TR: Diagnostic and Statistical Manual of Mental Disorders (5th ed.) Text Revision
VRET: Virtual Reality Exposure Therapy
GAD: Generalized Anxiety Disorder

## References

[1] American Psychiatric Association. Diagnostic and statistical manual of mental disorders (5th ed., text rev.; DSM-5-TR). 2022. 10.1176/appi.books.9780890425787.

[2] Oakes M, Bor R. The psychology of fear of flying (part II): a critical evaluation of current perspectives on approaches to treatment. Travel medicine and infectious disease. 2010;8(6):339–63. 10.1016/j.tmaid.2010.10.002 21071281

[3] Civil Aviation Authority. CAP 776 Global fatal accident review 1997–2006 (ISBN 978 0 11792 057 6). 2008. Retrieved February 4, 2025, from https://skybrary.aero/sites/default/files/bookshelf/502.pdf.

[4] Jones DR. Flying and danger, joy and fear. Aviat Space Environ Med. 1986;57(2):131–6. 3954701

[5] Maltby N, Kirsch I, Mayers M, Allen GJ. Virtual reality exposure therapy for the treatment of fear of flying: A controlled investigation. J Consult Clin Psychol. 2002;70(5):1112–8. 10.1037/0022-006X.70.5.1112. 12362961

[6] Curtis G, Magee WJ, Eaton WW, Wittchen HU, Kessler RC. Specific fears and phobias: Epidemiology and classification. Br J Psychiatry. 1998;173(3):212–7. 9926096

[7] Van Gerwen LJ, Spinhoven P, Van Dyck R. Behavioral and cognitive group treatment for fear of flying: A randomized controlled trial. J Behav Ther Exp Psychiatry. 2006;37(4):358–71. 10.1016/j.jbtep.2006.05.002. 16828460

[8] Oakes M, Bor R. The psychology of fear of flying (part I): a critical evaluation of current perspectives on the nature, prevalence and etiology of fear of flying. Travel Med Infect Dis. 2010;8(6):327–38. 10.1016/j.tmaid.2010.10.001. 21050826

[9] American Psychiatric Association. Diagnostic and statistical manual of mental disorders. 5th ed. Arlington: American Psychiatric Publishing; 2013.

[10] Coelho CM, Waters AM, Hine TJ, Wallis G. The use of virtual reality in acrophobia research and treatment. J Anxiety Disord. 2009;23(5):563–74. 10.1016/j.janxdis.2009.01.014. 19282142

[11] Hettema JM, Neale MC, Kendler KS. A review and meta-analysis of the genetic epidemiology of anxiety disorders. Am J Psychiatry. 2001;158(10):1568–78. 10.1176/appi.ajp.158.10.1568. 11578982

[12] Busscher B, Spinhoven P. Cognitive Coping as a Mechanism of Change in Cognitive-Behavioral Therapy for Fear of Flying: A Longitudinal Study With 3-Year Follow-Up. J Clin Psychol. 2017;73(9):1064–75. 10.1002/jclp.22424. 27983750

[13] Wilhelm FH, Roth WT. Clinical characteristics of flight phobia. J Anxiety Disord. 1997;11(3):241–61. 10.1016/S0887-6185(97)00009-1. 9220299

[14] Clark DA, Beck AT. Cognitive therapy of anxiety disorders: Science and practice. New York: Guilford Press; 2010.

[15] Starcevic V. Anxiety Disorders in Adults A Clinical Guide. Oxford university press; 2009.

[16] Galea S, Ahern J, Resnick H, Kilpatrick D, Bucuvalas M, Gold J, Vlahov D. Psychological sequelae of the September 11 terrorist attacks in New York City. N Engl J Med. 2002;346(13):982–7. 10.1056/NEJMsa013404. 11919308

[17] Ahern J, Galea S, Resnick H, Vlahov D. Television images and probable posttraumatic stress disorder after September 11: the role of background characteristics, event exposures, and perievent panic. J Nerv Ment Dis. 2004;192(3):217–26. 10.1097/01.nmd.0000116465.99830.ca. 15091303

[18] Bor R, Hubbard T, editors. Aviation Mental Health: Psychological Implications for Air Transportation. 1st ed. Routledge; 2006. 10.4324/9781315568560.

[19] Clark DM. A cognitive approach to panic. Behav Res Ther. 1986;24(4):461–70. 10.1016/0005-7967(86)90011-2. 3741311

[20] MacBlain S. Learning Theories for Early Years Practice. London: SAGE; 2021.

[21] Van Gerwen LJ, Diekstra RFW. Fear of flying treatment programs for passengers: An international review. Travel Med Infect Dis. 2000;6(5):355–60. 10.1016/j.tmaid.2004.01.002 17291954

[22] Vraga EK, Bode L. Using Expert Sources to Correct Health Misinformation in Social Media. Sci Communication. 2017;39(5):621–45. 10.1177/1075547017731776.

[23] Cheng JW, Mitomo H, Otsuka T, Jeon SY. Cultivation effects of mass and social media on perceptions and behavioural intentions in post-disaster recovery–The case of the 2011 Great East Japan Earthquake. Telematics Inform. 2016;33(3):753–72. 10.1016/j.tele.2015.12.001.

[24] Stein MB. Neurobiological perspectives on social phobia: from affiliation to zoology. Biol Psychiatry. 1998;44(12):1277–85. 10.1093/oso/9780195369250.001.0001. 9861470

[25] Van Gerwen LJG, Spinhoven P, Van Dyck R, Diekstra RFW. Construction and psychometric characteristics of two self-report questionnaires for the assessment of fear of flying. Psychol Assess. 1999;11(2):146–58. 10.1037/1040-3590.11.2.146.

[26] Ano GG, Vasconcelles EB. Religious coping and psychological adjustment to stress: A meta-analysis. J Clin Psychol. 2005;61(4):461–80. 10.1002/jclp.20049. https://onlinelibrary.wiley.com/doi/. 15503316

[27] Hofmann SG, Sawyer AT, Witt AA, Oh D. The effect of mindfulness-based therapy on anxiety and depression: A meta-analytic review. J Consult Clin Psychol. 2010;78(2):169–83. 10.1037/a0018555. 20350028 PMC2848393

[28] Davis M, Eshelman ER, McKay M. The relaxation and stress reduction workbook. New Harbinger Publications; 2008.

[29] Powers MB, Emmelkamp PM. Virtual reality exposure therapy for anxiety disorders: A meta-analysis. J Anxiety Disord. 2008;22(3):561–9. 10.1016/j.janxdis.2007.04.006. 17544252

[30] Rothbaum BO, Hodges L, Smith S, Lee JH, Price L. A controlled study of virtual reality exposure therapy for the fear of flying. J Consult Clin Psychol. 2000;68(6):1020–6. 10.1037/0022-006X.68.6.1020. 11142535

[31] Van Gerwen LJ, Diekstra RF, Arondeus JM, Wolfger R. Fear of flying treatment programs for passengers: An international update. Travel Med Infect Dis. 2004;2(1):27–35. 10.1016/j.tmaid.2004.01.002. 17291954

[32] Turkish Airlines. (n.d.). Fear of flying. Retrieved February 4, 2025, from; https://www.turkishairlines.com/en-int/flights/fly-different/fly-good-feel-good/before-flight/fear-of-flying.

[33] Lufthansa Aviation Training. Fear of flying seminar. Lufthansa Aviat Train. 2024. https://www.lufthansa-aviation-training.com/fear-of-flying-seminar.

[34] Lincoln YS. Naturalistic inquiry (Vol. 75). Sage; 1985.

[35] Sandelowski M. Real qualitative researchers do not count: The use of numbers in qualitative research. Res Nurs Health. 2001;24(3):230–40. 10.1002/nur.1025. 11526621

[36] Braun V, Clarke V. Reflecting on reflexive thematic analysis. Qualitative Res Sport Exerc Health. 2019;11(4):589–97. 10.1080/2159676X.2019.1628806.

[37] Lavrakas PJ. Encyclopedia of survey research methods. Sage publications; 2008.

[38] Sadler GR, Lee HC, Lim RSH, Fullerton J. Recruitment of hard-to-reach population subgroups via adaptations of the snowball sampling strategy. Nurs Health Sci. 2010;12(3):369–74. 10.1111/j.1442-2018.2010.00541.x. 20727089 PMC3222300

[39] Noy C. Sampling knowledge: The hermeneutics of snowball sampling in qualitative research. Int J Soc Res Methodol. 2008;11(4):327–44. 10.1080/13645570701401305.

[40] Guest G, Bunce A, Johnson L. How Many Interviews Are Enough? An Experiment with Data Saturation and Variability: An Experiment with Data Saturation and Variability. Field Methods. 2006;18(1):59–82. 10.1177/1525822X05279903.

[41] Braun V, Clarke V. Thematic analysis: A practical guide. Sage; 2021.

[42] Keenan SJ. Behind Social Media: A World of Manipulation and Control. Pop Culture Intersections. 2020. p. 48. Retrieved February 4, 2025, from https://scholarcommons.scu.edu/engl_176/48.

[43] Harvard Health Publishing. Understanding the stress response. Harvard Health; 2024. Retrieved June 21, 2026, from; https://www.health.harvard.edu/healthy-aging-and-longevity/understanding-the-stress-response.

[44] Ahmed SK, Mohammed RA, Nashwan AJ, Ibrahim RH, Abdalla AQ, Ameen BMM, Khdhir RM. Using thematic analysis in qualitative research. J Med Surg Public Health. 2025;6:100198.

[45] Wang W, Cole S, Chancellor C. Media’s impact on people’s anxiety levels toward air travel. 2010 TTRA International Conference. 2010. Retrieved from https://scholarworks.umass.edu/entities/publication/cb12f58f-6cdd-4ff0-b1d3-3a58a57bdc1d.

[46] Ferrara E, Yang Z. Measuring emotional contagion in social media. PLoS ONE. 2015;10(11):e0142390. 10.1371/journal.pone.0142390. 26544688 PMC4636231

[47] Bor R, van Gerwen LJ. Psychological perspectives on fear of flying. Ashgate Publishing Ltd. 2003. 10.4324/9781315245737.

[48] Beck AT, Clark DA. An information processing model of anxiety: Automatic and strategic processes. Behav Res Ther. 1997;35(1):49–58. 10.1016/S0005-7967(96)00069-1. 9009043

[49] Mineka S, Zinbarg R. A contemporary learning theory perspective on the etiology of anxiety disorders. Am Psychol. 2006;61(1):10–26. 10.1037/0003-066x.61.1.10. 16435973

[50] Gerbner G, Gross L, Morgan M, Signorielli N, Shanahan J. Growing up with television: Cultivation processes. In: Bryant J, Zillmann D, editors. Media effects: Advances in theory and research. 2nd ed. Lawrence Erlbaum Associates; 2002. p. 43–67.

[51] Johnston LG, Sabin K. Sampling hard-to-reach populations with respondent driven sampling. Methodological Innovations Online. 2010;5(2):38–48. 10.4256/mio.2010.0017.

